# Calibration of Priors for Bayesian Model‐Based Dose‐Finding Trial Designs With Joint Outcomes

**DOI:** 10.1002/sim.70746

**Published:** 2026-10-01

**Authors:** Emily Alger, Shing M. Lee, Ying Kuen K. Cheung, Christina Yap

**Affiliations:** ^1^ Clinical Trial and Statistics Unit Institute of Cancer Research London UK; ^2^ Department of Biostatistics Mailman School of Public Health, Columbia University New York New York USA

**Keywords:** Continual Reassessment Method, dose‐finding trials, joint outcomes, Phase I, prior calibration, priors

## Abstract

The goal of dose‐finding oncology trials is to assess the safety of anti‐cancer treatments across multiple doses and to recommend dose(s) for subsequent trials. As patients' outcomes accrue, trialists dynamically recommend new doses for further investigation during the trial. This adaptive decision‐making lends itself to Bayesian learning, with Bayesian frameworks increasingly guiding dose recommendations in model‐based dose‐finding designs, including the Continual Reassessment Method (CRM). However, such approaches introduce increased complexity, not least when additional outcomes are incorporated within designs. Such trial designs require careful prior selection. Directly applying prior calibration methods developed for single outcome model‐based trial designs to joint outcome trial designs may introduce unintended bias in dose recommendations, potentially limiting dose exploration and failing to accurately reflect trialists' a priori beliefs. We extend methodology to analytically calibrate priors for model‐based joint outcome trial designs with divergence minimisation. Our method offers an analytical and computationally efficient technique. We demonstrate the flexibility of our calibration method relative to existing approaches in ensemble simulation scenarios, and show that calibrating priors in this way delivers improved accuracy and computational efficiency compared with traditional grid search methods. As Bayesian dose‐finding trial designs continue to advance, research and guidance on the effective calibration of design parameters is essential to support uptake and ensure optimal performance in practice. This method provides an analytical and intuitive approach to prior calibration, highlighting the importance of rigorous prior calibration in improving model accuracy and dose selection for safer, more effective oncology treatments.

## 1 Introduction

Compared to rule‐based dose‐finding trial designs, Bayesian model‐based adaptive trial designs such as the Continual Reassessment Method (CRM) continue to gain popularity as flexible and efficient trial designs for determining recommended treatment doses in early‐phase dose‐finding trials [1, 2, 3, 4]. Bayesian dose‐finding designs commence with a prior probability distribution, and utilize accumulating patient data to inform a posterior probability distribution which is iteratively updated as patients' outcomes are observed to improve the accuracy of posterior estimates [2, 5]. The CRM trial design is a common model‐based dose‐finding trial design [4] and has been repeatedly shown to outperform rule‐based designs, such as the 3 + 3 design, in the majority of scenarios [6].

For traditional cytotoxic treatments, it is assumed that larger doses are associated with a greater probability of response. Thus, large doses which safeguard some toxicity threshold are recommended as a maximum tolerated dose (MTD), with toxicities identified by a clinician as at least ‘Severe’ recognized as dose‐limiting toxicities (DLTs) [7]. For the single outcome CRM design, the true DLT probability for each dose is often estimated using a one‐parameter model and the dose with a DLT rate closest to some target is identified as the MTD and often then recommended for further investigation within a Phase II trial.

While the CRM can be utilized under either a Bayesian or likelihood framework, a Bayesian framework allows for the estimation of a posterior distribution, offering innate uncertainty quantification in the MTD estimates and allowing historical data and clinical expertise through the prior distribution. The specification of this prior over parameters is a key component of traditional Bayesian methodology and is desirable for trialists wishing to incorporate existing knowledge within their trial. Conversely, a prior leading to no preference in MTD, which we refer to as a dose‐agnostic prior, may be advantageous for trialists with no prior knowledge who wish to leverage the flexibility provided by Bayesian dose‐finding trial designs by assuming each dose is equally likely to be identified as best a priori. Within a dose‐finding trial, we denote parameter priors chosen to reflect pretrial assumptions about the best dose as calibrated. Prior calibration techniques have been developed to calibrate dose‐agnostic priors for the single outcome CRM, calibrating both the parameter and dose skeleton utilized by the model [8, 9]. These calibrated priors continue to be recommended and deployed in contemporary dose‐finding trials employing the CRM design [10, 11, 12].

In line with a broader shift in regulatory and scientific perspectives, [13] dose‐finding trials are moving beyond the traditional goal of determining the MTD, toward optimizing dosage with respect to additional key outcomes such as pharmacokinetics, pharmacodynamics, clinical activity and patient‐reported outcomes (PROs) [14, 15]. Optimizing dose selection can include the identification of an optimal biological dose which utilizes both toxicity and efficacy data within a dose‐finding oncology trial (DFOT) [16]. Further, research increasingly advocates for the integration of PROs within DFOTs to enhance the determination of tolerable doses by capturing patient‐assessed tolerability of symptomatic adverse events and overall side effect impact [17, 18, 19, 20].

This new dose‐selection paradigm encourages the utilization of additional outcomes beyond DLT information alone to inform decision‐making. This is driving the development of innovative trial designs, including model‐based joint outcome CRM designs such as the PRO‐CRM [21, 22] and seamless CRM design presented by Wages and Tait utilizing both toxicity and efficacy data (hereafter identified as Eff+Tox‐CRM) [23]. Joint outcome dose‐finding designs utilize different decision‐making rules to single outcome dose‐finding designs and the repurposing of the existing prior calibration methods [8, 9] developed for single outcome designs for trial designs with joint (or more) outcomes can introduce unexpected bias, potentially limiting dose exploration. Such bias may be particularly unfavorable for treatments such as molecularly targeted agents, where an MTD is not identified in one third of trials, [24] and where nonmonotonic dose‐response relationships are often observed. As these settings may particularly benefit from investigation within a joint outcome trial design, [25] a prior which allows exploration across all relevant dose levels is vital.

While the calibration of priors for trial designs is often viewed through the lens of improving trial operating characteristics, choosing meaningful priors is also vital to ensure prior beliefs are principally incorporated within a trial utilizing a Bayesian framework. A major strength of Bayesian decision‐making lies in its explicit quantification of uncertainty in model estimates. Priors are not themselves quantities which can be ‘right’ or ‘wrong’, but rather a practitioner's quantification of their beliefs before commencing the trial which are iteratively updated with accumulating data to inform a posterior. As such, methodology to translate priors elicited from clinical teams to design‐specific parameter priors is essential to ensure valid Bayesian application in practice.

This motivates our work, providing methodology to calibrate priors for Bayesian model‐based dose‐finding trial designs with joint outcomes using divergence minimisation. We develop a general framework to support the calibration of parameter priors according to any chosen prior probability distribution. We then demonstrate this methodology through two joint outcome CRM designs which serve as case studies, [21, 23] with rationale and simulation results demonstrating the advantages of effective joint prior calibration. We also demonstrate the computational and accuracy advantages of this method over a traditional grid search, with suitable priors identified up to 4000 times faster.

## 2 Methods

In this section, we provide a general framework for the calibration of priors for model‐based joint outcome dose‐finding trial designs. Importantly, when a priori beliefs are elicited from trial teams, these beliefs are associated with a prior directly on the dose, referred to as priors on the *dose space*. In this section, we discuss how these priors can be translated to priors on a trial design's *parameter space*, which inform escalation and final dose recommendation decision‐making.

### 2.1 General Methodological Framework

The best dose for DFOTs has traditionally been considered as the MTD of treatment, however the definition of a best dose in recent years has been increasingly extended to consider additional outcomes such as efficacy, pharmacokinetics or PROs [14]. Model‐based dose‐finding designs wish to identify a best dose $d^{*}$ from a set of M ordered doses $𝒟=(d_{1},\ldots ,d_{M})$ under investigation, with $d_{1}<...<d_{M}$. Such designs are parametrised by a vector of design parameters θ∈Θ distributed according to a prior f before data is collected. This prior is itself parametrised by hyperparameters ϕ∈Φ, with the prior distribution dependent on them θ∼f(θ;ϕ). The best dose $d^{*}$ is defined by a decision rule ν:Θ→𝒟 such that $d^{*}:=\nu (\theta )$, with the minimum best dose selected when more than one dose attains the best criterion value. Consequently, identification of the best dose depends on design parameters θ which are influenced by ϕ. Identification of the best dose may be used to inform the dose‐selection decision for the next cohort of patients or to inform the selection of the final recommended dose.

#### 2.1.1 Linking the Parameter Space to Dose Space Using H‐Sets

Introduced by O'Quigley and Shen [26], H‐sets link the parameter space Θ for model‐based DFOT designs with the observed dose space 𝒟. H‐sets partition the parameter space, such that for a model with an estimated parameter value within a given region, a single dose corresponds to the best dose. While H‐sets are most commonly utilized in prior calibration for the CRM design, their usage can be generalized to any model‐based DFOT design, such that,

(1) $$\begin{array}{l} H_{d}=\{\theta \in \Theta :\nu (\theta )=d\}. \end{array}$$

In words, $H_{d}$ contains all the values of θ for which dose d is identified as the best dose. Since θ is random and follows a prior distribution determined by hyperparameters, these hyperparameters ensure θ a priori falls within specific H‐sets. The randomness of θ will result in a distribution over doses $\mu_{\phi }$ on the dose space via H‐sets, describing the probability a dose is identified as the best dose a priori given hyperparameters,

(2) $$\begin{array}{ll} \mu_{\phi }(d) & :={\mathbb{P}}_{\phi }(d^{*}=d)={\mathbb{P}}_{\phi }(\nu (\theta )=d)={\mathbb{P}}_{\phi }(\theta \in H_{d})\\ & \;=\int f(\theta ;\phi )\cdot 1\{\theta \in H_{d}\}\;d\theta , \end{array}$$

where 1{A} denotes the indicator function taking value 1 when event A occurs and value 0 otherwise. In Figure 1 we provide an illustrative example describing (2).

**FIGURE 1:**
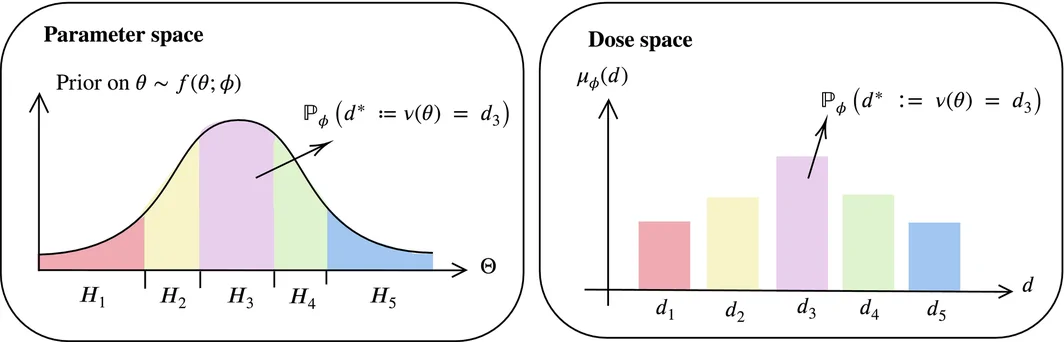
Illustrative example demonstrating how the parameter space Θ is partitioned into H‐sets $H_{1}$ to $H_{5}$ for a dose‐finding trial with 5 dose levels, $d_{1}$ to $d_{5}$. Each H‐set identifies the parameter values θ such that the best dose ν(θ) is recognized as a specific dose, with $\mu_{\phi }(d)$ quantifying the a priori probability dose d is the best dose for a prior with hyperparameters ϕ.

#### 2.1.2 Calibration of Hyperparameters

As $\mu_{\phi }$ denotes an a priori probability distribution P capturing a trial team's beliefs over the space of doses, our objective is to translate this belief into the parameter space by calibrating hyperparameters ϕ so $\mu_{\phi }$ reflects this elicited belief. To determine appropriate hyperparameters ϕ, one may measure the statistical divergence (distance) between $\mu_{\phi }$ and P. One exemplar divergence is the Kullback‐Leibler (KL) divergence, $D_{KL}(\mu_{\phi }||P)$ where

(3) $$\begin{array}{l} D_{KL}(\mu_{\phi }||P)=\underset{d\in D}{\sum }\mu_{\phi }(d)\log\frac{\mu_{\phi }(d)}{P(d)}. \end{array}$$

The KL divergence is a common metric for evaluating the statistical divergence between two distributions and has been utilized to inform selection of least informative priors for single outcome CRM designs [5]. The KL divergence is zero if and only if the two distributions P and $\mu_{\phi }$ are identical. Otherwise, the divergence is a nonnegative real‐number quantifying the likeness between the two distributions, with larger values indicating the two distributions are increasingly unlike.

To ensure $\mu_{\phi }$ best reflects the prior distribution P, we use the KL divergence as a calibration criterion to identify suitable hyperparameters $\phi^{*}\in \Phi$ that give a small divergence between $\mu_{\phi }$ and P,

(4) $$\begin{array}{l} \underset{d\in 𝒟}{\sum }\mu_{\phi }(d)\log\frac{\mu_{\phi }(d)}{P(d)}. \end{array}$$

This general framework supports the calibration of hyperparameters according to any well‐specified prior probability distribution P.

#### 2.1.3 Calibrating Dose‐Agnostic Priors

Calibrating priors to be dose‐agnostic is one common calibration approach [9] and assumes each dose is equally likely to be best a priori. This notion aligns with Lee and Cheung's [9] concept of a least informative prior variance, utilized to calibrate a prior based upon parameter variance for a single outcome CRM trial. Dose‐agnostic priors provide a simple and accessible calibration routine to support trialists in the identification of suitable priors for their Bayesian trial [9]. By supposing each dose is equally likely to be the MTD a priori, a dose‐agnostic calibration schema promotes the selection of a robust prior to ensure good operating performance across many potential trial scenarios.

To calibrate dose‐agnostic priors, the prior probability distribution P is chosen to be discrete uniform distribution and, supposing M doses are investigated, the hyperparameters ϕ are calibrated to ensure $\mu_{\phi }(d)=\frac{1}{M}\forall d\in 𝒟$ a priori. In this instance, the calibration criterion becomes,

(5) $$\begin{array}{l} \underset{d\in 𝒟}{\sum }\mu_{\phi }(d)\log(M\mu_{\phi }(d)). \end{array}$$

### 2.2 Prior Calibration for Model‐Based Joint Outcome Trial Designs

For joint outcome trial designs, the decision rule ν depends on more than one model parameter, with each parameter governed by its own set of hyperparameters. In this setting, $\mu_{\phi }(d)$ must be evaluated jointly and the KL divergence must be jointly optimized with respect to multiple sets of hyperparameters. In the following sections, we calibrate hyperparameters for two model‐based DFOT designs utilizing this calibration framework. For conciseness, we focus on the prior calibration for two trial designs which use a CRM backbone for modeling and seek to calibrate dose‐agnostic priors a priori, however other model‐based designs and prior distributions can also be explored using the framework detailed above.

The PRO‐CRM [21] and Eff+Tox‐CRM [23] designs make dosing decisions by determining the clinician‐assessed MTD with an additional component, whether that be a patient‐assessed MTD or the most effective dose respectively. In both instances, the joint dose recommendation depends on the distribution of the marginal dose recommendations for each endpoint and recommends the minimal of the two recommended doses. While the clinician‐assessed MTD is similarly identified within the PRO‐CRM and Eff+Tox‐CRM designs, the following sections outline two design‐specific approaches that ensure the resulting joint dose recommendations align with prespecified prior distributions.

#### 2.2.1 Motivating Example: Dose‐Agnostic Priors

The PRO‐CRM and Eff+Tox‐CRM consider two independent decision‐rules to recommend best doses $d_{1}^{*}$ and $d_{2}^{*}$ using each endpoint in turn. The final recommended dose is then defined as $d^{*}=\min(d_{1}^{*},d_{2}^{*})$. Dose‐agnostic priors are a common prior choice in Bayesian single outcome CRM trials and previous work for the PRO‐CRM and Eff+Tox‐CRM [22, 23] has considered calibrating parameter priors such that the prior distribution of $d_{1}^{*}$ and $d_{2}^{*}$ are marginally dose‐agnostic (i.e., a priori dose selection for each endpoint follows independent discrete uniform distributions). However this does not reflect a *jointly* dose‐agnostic prior, instead embedding a more conservative, risk‐averse prior within dose decision‐making; contrary to the trialist's intent of a jointly dose‐agnostic prior.

Consider a trial with d∈{1,...,M} doses. Suppose the parameter priors are calibrated to ensure the prior distribution over $d_{1}^{*}$ and $d_{2}^{*}$ are marginally dose‐agnostic. In this case, the probability mass function of the joint dose decision is,

(6) $$\begin{array}{l} \mathbb{P}(\min(d_{1}^{*},d_{2}^{*})=d)=\frac{2(M-d)+1}{M^{2}}. \end{array}$$

While the probability distribution of $d_{1}^{*}$ and $d_{2}^{*}$ are dose‐agnostic marginally, the joint decision is now left‐skewed compared to the scenario where dose‐agnostic priors are calibrated jointly to ensure $\mathbb{P}(\min(d_{1}^{*},d_{2}^{*})=d)=\frac{1}{M}\;\forall d$ a priori. This is made clear by considering the ratio between the a priori probability dose d+1 is the best dose compared to dose d,

(7) $$\begin{array}{l} \frac{\mathbb{P}(\min(d_{1}^{*},d_{2}^{*})=d+1)}{\mathbb{P}(\min(d_{1}^{*},d_{2}^{*})=d)}=\frac{2(M-d)-1}{2(M-d)+1}<1, \end{array}$$

demonstrating that the probability mass function of the joint dose‐selection decision is decreasing. As per (7), marginally calibrated priors assign more mass to smaller doses and less mass to larger doses (see Section S1 of the Supporting Information). Supposing jointly calibrated priors can be calibrated, it holds that for any trial with M doses, for any dose d such that

(8) $$\begin{array}{l} d>\frac{M+1}{2}, \end{array}$$

priors calibrated marginally will assign less mass to d than our jointly calibrated dose‐agnostic prior before even seeing any data.

As such, employing this marginally calibrated prior under the incorrect assumption that it is dose‐agnostic may unduly limit dose exploration within the trial by inadvertently favoring lower doses, unbeknownst to the trialist. By optimizing (5), we can find jointly calibrated dose‐agnostic priors ensuring a discrete uniform distribution over doses. This result holds for any trial design in which its joint dose‐selection decision considers the minimum of two (or more) marginal dose recommendations.

## 3 Continual Reassessment Method for Clinician‐ and Patient‐Reported Toxicity (PRO‐CRM)

In this section, we provide a calibration technique to jointly calibrate priors for the PRO‐CRM. The Bayesian extension to the PRO‐CRM trial design [21, 22] jointly considers clinician‐ and patient‐assessed DLTs to find a dose which is both admissible from a clinician and patient perspective. The binary endpoint $Y_{C}=1$ indicates an observed clinician‐assessed DLT (C‐DLT) within the DLT assessment window. Similarly, the binary endpoint $Y_{P}=1$ indicates an observed patient‐assessed DLT (P‐DLT) within the prespecified DLT assessment window.

Similarly to the CRM design [1], for d∈𝒟, the probabilities of C‐DLT and P‐DLT are estimated using a power model likelihood,

(9) $$\begin{array}{l} \pi_{C,d}=\mathbb{P}(Y_{C}=1|d)={v_{d}}^{\lambda }\;\text{and}\;\pi_{P,d}=\mathbb{P}(Y_{P}=1|d)={w_{d}}^{\xi }, \end{array}$$

where $v_{d}$ and $w_{d}$ are the prior probabilities (the ‘skeletons’) for C‐DLT and P‐DLT respectively. Further methodological details of the PRO‐CRM are provided in Section S2 of the Supporting Information.

For this trial design, θ=(λ,ξ) are the parameters defining the dose‐selection decision‐rule ν(θ), with associated hyperparameters $\phi =(\phi_{\lambda },\phi_{\xi })$ respectively. For $\hat{\lambda }$ and $\hat{\xi }$ the posterior mean point estimates of parameters λ and ξ respectively, the next dose $d^{*}$ is recommended using the rule,

(10) $$\begin{array}{l} \nu (\theta )=\min\{\nu_{C}(\lambda ),\nu_{P}(\xi )\}, \end{array}$$

where

(11) $$\begin{array}{l} \nu_{C}(\lambda )=\arg\underset{d\in 𝒟}{\min}|v_{d}^{\hat{\lambda }}-p_{C}|\;\text{and}\;\nu_{P}(\xi )=\arg\underset{d\in 𝒟}{\min}|w_{d}^{\hat{\xi }}-p_{P}|, \end{array}$$

with $p_{C}$ and $p_{P}$ indicating C‐DLT and P‐DLT target rates respectively. This is a conservative joint decision rule that selects the more cautious of the two marginal MTD recommendations based on C‐DLT and P‐DLT data.

### 3.1 Prior Calibration for PRO‐CRM

As per O'Quigley and Shen [26], the parameter space for λ and ξ can be partitioned into disjoint H‐sets in which specific doses are identified as best. For example, for parameter λ, the H‐sets ${\text{H}}_{\lambda }=\{H_{\lambda ,1},...,H_{\lambda ,M}\}$ are defined such that,

(12) $$\begin{array}{l} H_{\lambda ,d}=\{\lambda \in \Lambda :|{v_{d}}^{\lambda }-p_{C}|<|{v_{j}}^{\lambda }-p_{C}|\;\text{for}\;j\neq d\}. \end{array}$$

As H‐sets are disjoint from one another, ${\text{H}}_{\lambda }$ can be written as ${\text{H}}_{\lambda }=\{[a_{1},a_{2}),\ldots ,(a_{M},a_{M+1}]\}$. Using Cheung and Chappell [8], the boundaries $a_{d}$ can be found by solving,

(13) $$\begin{array}{l} {v_{d-1}}^{a_{d}}+{v_{d}}^{a_{d}}=2p_{C}. \end{array}$$

We can similarly construct H‐sets for ξ and can write $H_{\xi }=\{H_{\xi ,1},\ldots ,H_{\xi ,M}\}$ where ${\text{H}}_{\xi }=\{[b_{1},b_{2}),\ldots ,(b_{M},b_{M+1}]\}$.

As per Section 2.2.1, existing calibration approaches for the PRO‐CRM follow calibration recommendations provided by Lee and Cheung [9] for single outcome CRM designs with parameters λ and ξ marginally calibrated for a prior probability distribution P. In this instance, $\phi_{\lambda }$ and $\phi_{\xi }$ are calibrated independently with calibration criteria,

(14) $$\begin{array}{ll} & \underset{d\in 𝒟}{\sum }{\mathbb{P}}_{\phi_{\lambda }}(\nu_{C}(\lambda )\in H_{\lambda ,d})\log\left(\frac{{\mathbb{P}}_{\phi_{\lambda }}(\nu_{C}(\lambda )\in H_{\lambda ,d})}{P(d)}\right)\;\text{and}\\ & \;\underset{d\in 𝒟}{\sum }{\mathbb{P}}_{\phi_{\xi }}(\nu_{P}(\xi )\in H_{\xi ,d})\log\left(\frac{{\mathbb{P}}_{\phi_{\xi }}(\nu_{P}(\xi )\in H_{\xi ,d})}{P(d)}\right). \end{array}$$

However, the PRO‐CRM utilizes a joint decision rule (choosing the minimum of two marginally independent dose recommendations) which introduces a structural dependency which is not accounted for when λ and ξ are calibrated independently as per (14). Evaluation of a priori dose‐decision‐making under these marginally calibrated priors demonstrates a systematic bias toward lower‐dose recommendations–empirically demonstrating that one cannot assume marginally calibrated priors necessarily lead to similar behavior for model‐based joint outcome trial designs.

Accordingly, rather than selecting $\phi_{\lambda }$ and $\phi_{\xi }$ marginally to calibrate priors for the PRO‐CRM, we must calibrate priors $\phi =(\phi_{\lambda },\phi_{\xi })$ jointly. Assuming decision‐rules $\nu_{C}(\lambda )$ and $\nu_{P}(\xi )$ are constructed independently for simplicity as per (11), $\mu_{\phi }(d)$ can be written as follows,

(15) $$\begin{array}{cc} \mu_{\phi }(d) & ={\mathbb{P}}_{\phi }\left(\min\{\nu_{C}(\lambda ),\nu_{P}(\xi )\}=d\right)\\ & ={\mathbb{P}}_{\phi }\left(\min\{\nu_{C}(\lambda ),\nu_{P}(\xi )\}\geq d\right)\\ & \;-{\mathbb{P}}_{\phi }\left(\min\{\nu_{C}(\lambda ),\nu_{P}(\xi )\}\geq d+1\right)\\ & ={\mathbb{P}}_{\phi }\left(\nu_{C}(\lambda )\geq d\right)\;{\mathbb{P}}_{\phi }\left(\nu_{P}(\xi )\geq d\right)\\ & \;-{\mathbb{P}}_{\phi }\left(\nu_{C}(\lambda )\geq d+1\right)\;{\mathbb{P}}_{\phi }\left(\nu_{P}(\xi )\geq d+1\right)\\ & ={\mathbb{P}}_{\phi }\left(\lambda \in (a_{d},a_{M+1}]\right)\;{\mathbb{P}}_{\phi }\left(\xi \in (b_{d},b_{M+1}]\right)\\ & \;-{\mathbb{P}}_{\phi }\left(\lambda \in (a_{d+1},a_{M+1}]\right)\;{\mathbb{P}}_{\phi }\left(\xi \in (b_{d+1},b_{M+1}]\right). \end{array}$$

We note that $\mu_{\phi }(d)$ can be written in terms of the marginal dose recommendations $\nu_{C}(\lambda )$ and $\nu_{P}(\xi )$, allowing us to analytically define $\mu_{\phi }(d)$ with H‐sets using (13). Thus, for F and G denoting the cumulative distribution functions for λ and ξ with hyperparameters $\theta_{\lambda }$ and $\theta_{\xi }$ respectively,

(16) $$\begin{array}{c} \mu_{\phi }(d)=\left\{\begin{array}{ll} 1-(1-F(a_{d+1};\phi_{\lambda }))(1-G(b_{d+1};\phi_{\xi }))\; & \text{if}\;d=1,\\ (1-F(a_{d};\phi_{\lambda }))(1-G(b_{d};\phi_{\xi }))\; & \\ \;-\;(1-F(a_{d+1};\phi_{\lambda }))(1-G(b_{d+1};\phi_{\xi }))\; & \text{if}\;1<d<M,\\ (1-F(a_{d};\phi_{\lambda }))(1-G(b_{d};\phi_{\xi }))\; & \text{if}\;d=M. \end{array}\right. \end{array}$$

As $\mu_{\phi }(d)$ has an analytical form, the KL divergence between $\mu_{\phi }(d)$ and the prior probability distribution P can be evaluated directly as per (5), and optimisation functions such as optim in R can be used to find suitable parameter values in a computationally efficient manner (see Section 5.1). Methodology can be further extended to incorporate dependence between dose recommendations induced by $\nu_{C}$ and $\nu_{P}$ with a copula, further discussed in the Discussion.

### 3.2 PRO‐CRM Simulation Set‐up

To demonstrate the joint calibration of priors for the PRO‐CRM trial and their role in interim and final decision‐making, we present a simulation study. While methodology presented in the preceding section can be utilized to jointly calibrate priors according to any prior distribution P, for this specific study we compare a PRO‐CRM trial in which priors are marginally calibrated to be dose‐agnostic with another trial where priors are jointly calibrated to be dose‐agnostic as per Section 3.1.

We consider PRO‐CRM in a trial with five increasing doses with a sample size of 39 patients sequentially enrolled in cohorts of three. DLTs are all observed before the next cohort of patients are enrolled. Target C‐DLT rate $p_{C}$ and P‐DLT rate $p_{P}$ are set to 0.25 and 0.35 respectively. Correlated C‐DLT and P‐DLT outcomes are simulated using a Clayton copula with Cox‐exponential proportional hazard survival models simulating time‐to‐DLT. Parameter φ controls correlation, and is set to φ=0.9 to introduce moderate correlation (equivalent to Kendall's Tau τ=0.31). More details are provided in the simulation schema for U‐PRO‐CRM, a utility‐based decision‐making adaptation for PRO‐CRM [27].

We calibrate C‐DLT and P‐DLT skeletons associated with the C‐DLT and P‐DLT rates as (0.06, 0.14, 0.25, 0.38, 0.50) and (0.10, 0.21, 0.35, 0.49, 0.61) respectively as per Lee et al. [28] using R package dfcrm [29].

#### 3.2.1 Marginally Calibrated Priors

Both lognormal and gamma distributions have traditionally been used as priors for Bayesian CRM modeling. In this paper, we explore gamma priors which provide us with increased flexibility for calibrating priors. For a sample size of 39 patients with skeletons as per Lee et al. [21], priors calibrated marginally using existing methodology for a single outcome CRM are,

(17) $$\begin{array}{l} \lambda_{M}∼\text{Gamma}(2.78,2.37)\;\text{and}\;\xi_{M}∼\text{Gamma}(2.34,1.96). \end{array}$$

These priors are subsequently identified as the ‘Marginally calibrated’ prior throughout this paper and may be identified as the ‘Marginal recommendation’ within the proceeding simulation study. Figure 4 demonstrates a priori dose recommendations when priors are marginally calibrated for PRO‐CRM.

#### 3.2.2 Dose‐Agnostic Priors

In Section 3.1 we present methodology to calibrate priors according to any prespecified prior choice. To detail the impact of prior calibration on trial design performance, we jointly calibrate priors for the PRO‐CRM and Eff+Tox‐CRM case study within this paper to be dose‐agnostic.

Considering the prior $\lambda_{J}∼\text{Gamma}(\alpha_{\lambda },\beta_{\lambda })$ and $\xi_{J}∼\text{Gamma}(\alpha_{\xi },\beta_{\xi })$, to jointly calibrate parameter priors for the PRO‐CRM we use calibration criterion (5) over $\phi =(\alpha_{\lambda },\beta_{\lambda },\alpha_{\xi },\beta_{\xi })$. Supposing we wish to calibrate dose‐agnostic priors, the optimisation of $D_{KL}(\mu_{\phi }|\text{Uniform})$ allows for infinitely many solutions between two cases–where either the Clinician‐CRM or Patient‐CRM always recommend dose $d_{M}$ a priori. In these instances, best dose recommendations for one endpoint will only recommend the largest dose $d_{M}$ and the prior on the other endpoint can be calibrated using the traditional dose‐agnostic marginal methodology for single outcome CRM designs. To find suitable hyperparameters associated with a small $D_{KL}(\mu_{\phi }|\text{Uniform})$ between these cases, we fix one hyperparameter $\beta_{\xi }$ and optimize the other hyperparameter values.

Figure 2 demonstrates the relationship between endpoint dose recommendations for varying $\beta_{\xi }$ under this set‐up. This figure demonstrates the trade‐off between Clinician‐CRM (using C‐DLT data alone to inform the best dose) and Patient‐CRM (using P‐DLT data alone to inform the best dose) divergence to jointly calibrate dose‐agnostic priors for PRO‐CRM. Following simulations, we find that the choice of $\beta_{\xi }$ (and subsequent selection of dose‐agnostic priors) does not significantly impact simulation results. As such, we choose $\beta_{\xi }$ such that the KL divergence for the Clinician‐CRM and Patient‐CRM is equal. The marginal KL divergence for these priors is 0.17 for both the Clinician‐CRM and Patient‐CRM.

**FIGURE 2:**
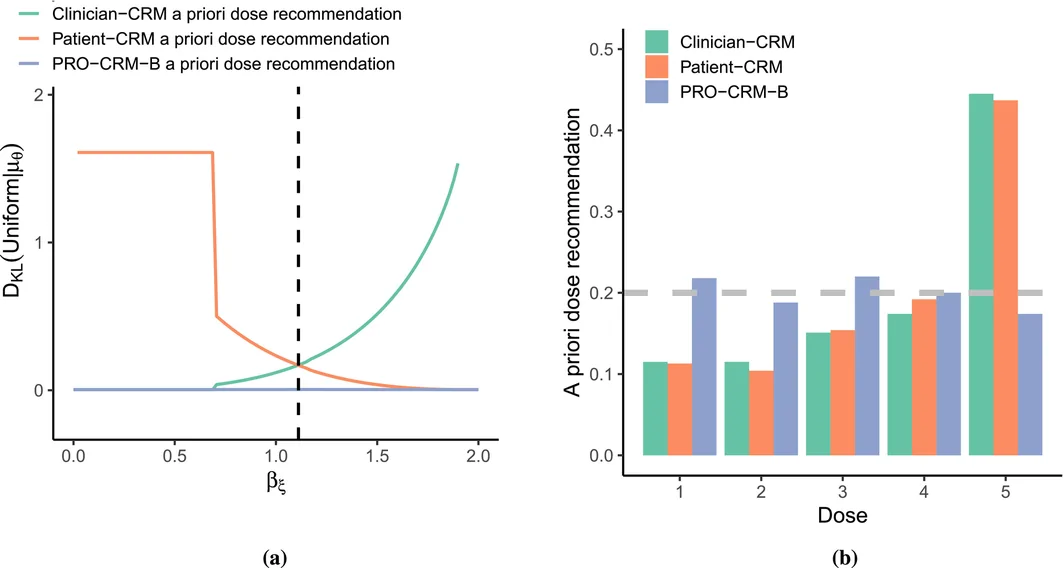
Comparison of a priori dose recommendations for PRO‐CRM a priori MTD, Clinician‐CRM MTD and Patient‐CRM MTD. A total of 39 patients are treated in cohorts of 3. (a) KL divergence for a priori dose selections for Clinician‐CRM, Patient‐CRM and PRO‐CRM with a dashed line indicating $\beta_{\xi }=1.11.$ (b) 1,000 simulated a priori dose recommendations with $\beta_{\xi }=1.11$ for Clinician‐CRM, Patient‐CRM and PRO‐CRM.

As such we select priors,

(18) $$\begin{array}{l} \lambda_{J}∼\;\text{Gamma}(2.42,1.35)\;\text{and}\;\xi_{J}∼\;\text{Gamma}(2.09,1.11). \end{array}$$

A simulation of a priori dose recommendations for these parameters is presented in Figure 2.

#### 3.2.3 Hypothetical Trial Example for PRO‐CRM

To illustrate the role of prior calibration on trial performance, we detail a hypothetical Phase I trial employing PRO‐CRM with marginally calibrated priors (17) and dose‐agnostic priors (18). The trial is simulated as per Section 3.2 with probability of C‐ and P‐DLT (0.02, 0.05, 0.10, 0.20, 0.25) and (0.09, 0.17, 0.20, 0.25, 0.35) for doses respectively. P‐DLTs and C‐DLTs are simulated as per Cheung [30] to generate a complete outcome profile for each patient in the trial such that their outcomes can be simulated for every dose. Under this scenario, dose 5 is the MTD, necessitating both Clinician‐MTD and Patient‐MTD be independently identified as dose 5 and thus requiring $\lambda \in H_{\lambda ,5}$ and $\xi \in H_{\xi ,5}$.

In Figure 3, we follow the allocation of patients to each dose and detail the estimation of exponents determining C‐DLT and P‐DLT rates with DLT responses for observed patients under each prior calibration using marginally calibrated priors (17) and jointly calibrated priors (18). Figures 3b and 3c present the cohorts for which C‐DLT and P‐DLT parameters lie in dose 5's H‐set for each respective prior calibration. When jointly dose‐agnostic priors are calibrated, dose 5 is persistently recognized as the MTD. From Cohort 5 onwards, all patients are allocated to dose 5 until the end of the trial and this dose is subsequently identified as best. However, when priors are marginally calibrated, dose 5 is only evaluated in cohorts 5 and 6 before being incorrectly deemed overly toxic, after which all subsequent patient cohorts are assigned to dose 4. There is never again an instance where C‐DLT and P‐DLT exponents lie within the dose 5's H‐set and dose 4 is subsequently incorrectly identified as the MTD at the end of the trial.

**FIGURE 3:**
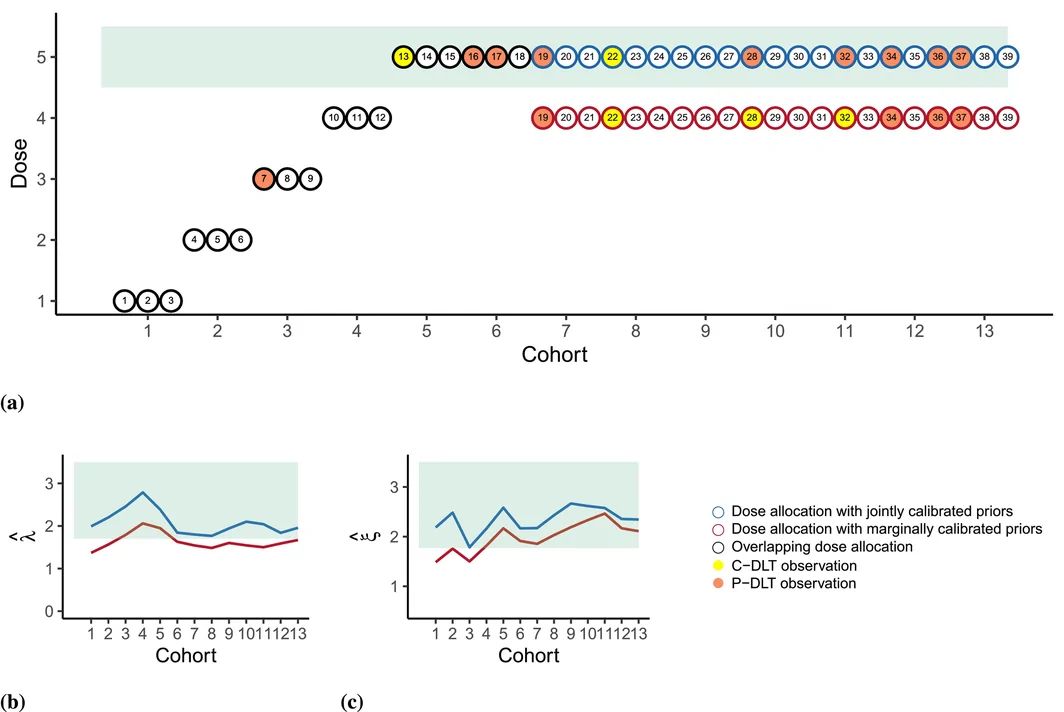
Hypothetical dose‐finding trial using PRO‐CRM with true C‐DLT rates (0.02, 0.05, 0.10, 0.20, 0.25) and true P‐DLT rates (0.09, 0.17, 0.20, 0.25, 0.35) for marginally calibrated priors shown in red (17) and the trial with jointly calibrated dose‐agnostic priors shown in blue (18). A total of 39 patients are treated in cohorts of 3. (a) Recommended doses at each interim analysis. Dose 5, marked in green, is the true MTD. Patients 16, 17, 19 experience both a C‐DLT and P‐DLT, and patients 32, 34, 36 experience both C‐DLT and P‐DLT when assigned to dose 5. (b) $\hat{\lambda }$ at each interim analysis with the H‐set for the MTD (dose 5) highlighted in green, (c) $\hat{\xi }$ at each interim analysis with the H‐set for the MTD (dose 5) highlighted in green.

#### 3.2.4 Ensemble Simulation Study

We demonstrate the performance of the PRO‐CRM with different prior calibrations in an ensemble simulation study [31]. This simulation study evaluates the performance of PRO‐CRM across all possible configurations of dose‐toxicity relationship for C‐DLTs and P‐DLTs. Under this trial design, six clinician‐ and patient‐MTD configurations are possible, corresponding to dose levels 1–5 as the MTD or the case in which no MTD is identified. We therefore investigate $6^{2}=36$ possible simulation scenarios, with 1250 trials simulated for each scenario in which we compare marginally calibrated and dose‐agnostic priors. In Section S3 of the Supporting Information, we document all 36 investigated simulation scenarios. Dose‐agnostic priors suppose that a priori each dose is assumed to be equally likely to be the MTD. In this case, we would anticipate that PCS is similar across simulation scenarios in comparison to an alternate prior which instead assigns more prior mass to one dose above another. To demonstrate this, we evaluate the performance of these calibrated priors by considering the standard deviation of the probability of correct selection (PCS) across all 36 scenarios. Reporting the standard deviation of PCS indicates the stability of PCS across scenarios which consider all doses as the MTD in turn. As well as reporting the average (mean) PCS and PCS standard deviation across all simulation scenarios, we also present twelve random scenarios from the 36 scenarios investigated in this ensemble simulation study in Section 3.3 to demonstrate the impact of different prior selections on scenario‐specific PCS. Simulation results of all 36 scenarios can be found at https://github.com/alemily100/calibrate_priors.

### 3.3 PRO‐CRM Results

Across all 36 scenarios, utilizing PRO‐CRM with dose‐agnostic priors reduces the standard deviation of PCS compared to marginally calibrated priors while maintaining mean PCS performance. Specifically, the standard deviation of PCS decreases from 0.18 under marginally calibrated priors to 0.14 under dose‐agnostic priors, indicating that dose‐agnostic priors yield more consistent and robust PCS performance across all scenarios. Importantly, this reduction in variability does not come at the expense of mean PCS, with dose‐agnostic priors achieving a slightly higher mean PCS across all scenarios of 0.60 compared to 0.58 when the PRO‐CRM utilizes marginally calibrated priors.

Table 1 presents mean PCS aggregated by the true MTD across all ensemble scenarios. As dose‐agnostic priors substantially improve PCS (up to 13%) when the MTD is the largest dose, and maintain equivalent performance (within 1%) when the MTD is at lower doses, dose‐agnostic priors achieve a reduced standard deviation of PCS compared to a trial with marginally calibrated priors.

**TABLE 1:** Average PCS across all 36 ensemble simulation scenarios for the PRO‐CRM, aggregated by the true MTD for each scenario under marginally calibrated and jointly calibrated dose‐agnostic priors.

	Dose level				
	1	2	3	4	5
**PCS under marginally calibrated priors**	0.77	0.62	0.56	0.41	0.25
**PCS under dose‐agnostic priors**	0.76	0.61	0.57	0.46	0.38

The specific advantages of dose‐agnostic priors are further demonstrated in Table 2, which illustrates PCS performance for 12 randomly generated simulation scenarios under each prior choice. The robustness of dose‐agnostic priors for PCS is most evident in Scenario 1. In this scenario, dose 5 is best and using dose‐agnostic priors increases the probability of correctly identifying the MTD by 15% relative to marginally calibrated priors, bringing the PCS for higher‐dose MTDs more in line with those observed for lower‐dose MTDs.

**TABLE 2:** Proportion of MTD selection recommendations for each dose under 12 random scenarios for PRO‐CRM with marginally (17) and jointly calibrated priors (18). Bold indicates the best dose according to C‐DLT or P‐DLTs only. Green indicates the best joint dose decision as per PRO‐CRM.

		Dose level				
		1	2	3	4	5
**Scenario 1**	Probability of Clinician‐DLT	0.05	0.10	0.12	0.15	**0.20**
	Probability of Patient‐DLT	0.04	0.09	0.17	0.20	**0.25**
	Marginal recommendation	0.00	0.01	0.10	0.47	0.42
	Dose‐agnostic recommendation	0.00	0.00	0.07	0.36	0.57
**Scenario 2**	Probability of Clinician‐DLT	0.05	0.10	0.12	0.15	**0.20**
	Probability of Patient‐DLT	0.20	0.25	**0.35**	0.50	0.65
	Marginal recommendation	0.02	0.25	0.60	0.13	0.00
	Dose‐agnostic recommendation	0.02	0.20	0.63	0.15	0.00
**Scenario 3**	Probability of Clinician‐DLT	0.10	0.12	0.15	0.20	**0.25**
	Probability of Patient‐DLT	0.25	**0.35**	0.50	0.65	0.80
	Marginal recommendation	0.16	0.64	0.20	0.00	0.00
	Dose‐agnostic recommendation	0.15	0.62	0.23	0.00	0.00
**Scenario 4**	Probability of Clinician‐DLT	0.12	0.15	0.20	**0.25**	0.40
	Probability of Patient‐DLT	0.09	0.17	0.20	0.25	**0.35**
	Marginal recommendation	0.01	0.13	0.44	0.38	0.03
	Dose‐agnostic recommendation	0.01	0.10	0.42	0.41	0.06
**Scenario 5**	Probability of Clinician‐DLT	0.12	0.15	0.20	**0.25**	0.40
	Probability of Patient‐DLT	**0.35**	0.50	0.65	0.80	0.85
	Marginal recommendation	0.74	0.26	0.00	0.00	0.00
	Dose‐agnostic recommendation	0.73	0.26	0.00	0.00	0.00
**Scenario 6**	Probability of Clinician‐DLT	0.15	0.20	**0.25**	0.40	0.55
	Probability of Patient‐DLT	0.04	0.09	0.17	0.20	**0.25**
	Marginal recommendation	0.04	0.30	0.53	0.12	0.00
	Dose‐agnostic recommendation	0.04	0.23	0.57	0.15	0.00
**Scenario 7**	Probability of Clinician‐DLT	0.15	0.20	**0.25**	0.40	0.55
	Probability of Patient‐DLT	0.17	0.20	0.25	**0.35**	0.50
	Marginal recommendation	0.05	0.34	0.52	0.09	0.00
	Dose‐agnostic recommendation	0.05	0.29	0.56	0.10	0.00
**Scenario 8**	Probability of Clinician‐DLT	0.15	0.20	**0.25**	0.40	0.55
	Probability of Patient‐DLT	0.25	**0.35**	0.50	0.65	0.80
	Marginal recommendation	0.20	0.66	0.15	0.00	0.00
	Dose‐agnostic recommendation	0.20	0.63	0.16	0.00	0.00
**Scenario 9**	Probability of Clinician‐DLT	0.20	**0.25**	0.40	0.55	0.60
	Probability of Patient‐DLT	0.09	0.17	0.20	0.25	**0.35**
	Marginal recommendation	0.23	0.58	0.19	0.00	0.00
	Dose‐agnostic recommendation	0.19	0.58	0.23	0.00	0.00
**Scenario 10**	Probability of Clinician‐DLT	0.20	**0.25**	0.40	0.55	0.60
	Probability of Patient‐DLT	0.17	0.20	0.25	**0.35**	0.50
	Marginal recommendation	0.22	0.60	0.18	0.00	0.00
	Dose‐agnostic recommendation	0.21	0.60	0.18	0.00	0.00
**Scenario 11**	Probability of Clinician‐DLT	**0.25**	0.40	0.55	0.60	0.70
	Probability of Patient‐DLT	0.20	0.25	**0.35**	0.50	0.65
	Marginal recommendation	0.75	0.25	0.00	0.00	0.00
	Dose‐agnostic recommendation	0.74	0.26	0.00	0.00	0.00
**Scenario 12**	Probability of Clinician‐DLT	**0.25**	0.40	0.55	0.60	0.70
	Probability of Patient‐DLT	0.25	**0.35**	0.50	0.65	0.80
	Marginal recommendation	0.77	0.22	0.00	0.00	0.00
	Dose‐agnostic recommendation	0.77	0.22	0.00	0.00	0.00

In Table 3 we detail the proportion of patients correctly dosed, underdosed and overdosed across all scenarios and the 12 random scenarios presented in Table 2. Utilizing dose‐agnostic priors reduces the proportion of patients underdosed across every scenario. When the true MTD is higher, dose‐agnostic priors increase the number of patients assigned to the MTD, most evidently in Scenario 1 where this is dose 5. In this scenario, dose‐agnostic priors improve patient allocation to the MTD by 11%. For other scenarios, the proportion of patients assigned to the MTD is comparative between the dose‐agnostic and marginally calibrated priors. Utilizing dose‐agnostic priors overdoses slightly more (up to 6%) of patients compared to a conservative trial which utilizes marginally calibrated priors.

**TABLE 3:** Proportion of patients correctly dosed, underdosed or overdosed for each random scenario for the PRO‐CRM design with marginally and jointly calibrated priors.

		Best dose	Underdosed	Overdosed
**Across all ensemble scenarios**	Marginal prior	**0.24**	0.47	0.30
	Dose‐agnostic prior	0.22	0.42	0.36
**Scenario 1 [MTD at Dose 5]**	Marginal prior	0.19	0.81	0.00
	Dose‐agnostic prior	**0.30**	0.70	0.00
**Scenario 2 [MTD at Dose 3]**	Marginal prior	**0.45**	0.43	0.12
	Dose‐agnostic prior	0.44	0.39	0.17
**Scenario 3 [MTD at Dose 2]**	Marginal prior	**0.50**	0.24	0.25
	Dose‐agnostic prior	0.49	0.23	0.29
**Scenario 4 [MTD at Dose 4]**	Marginal prior	0.26	0.71	0.03
	Dose‐agnostic prior	**0.28**	0.66	0.06
**Scenario 5 [MTD at Dose 1]**	Marginal prior	**0.62**	0.00	0.38
	Dose‐agnostic prior	0.58	0.00	0.42
**Scenario 6 [MTD at Dose 3]**	Marginal prior	**0.42**	0.45	0.14
	Dose‐agnostic prior	0.40	0.40	0.19
**Scenario 7 [MTD at Dose 3]**	Marginal prior	0.38	0.53	0.08
	Dose‐agnostic prior	**0.39**	0.47	0.14
**Scenario 8 [MTD at Dose 2]**	Marginal prior	**0.51**	0.29	0.21
	Dose‐agnostic prior	0.50	0.27	0.23
**Scenario 9 [MTD at Dose 2]**	Marginal prior	**0.47**	0.27	0.26
	Dose‐agnostic prior	0.44	0.25	0.31
**Scenario 10 [MTD at Dose 2]**	Marginal prior	0.46	0.29	0.25
	Dose‐agnostic prior	**0.48**	0.26	0.27
**Scenario 11 [MTD at Dose 1]**	Marginal prior	**0.60**	0.00	0.40
	Dose‐agnostic prior	0.59	0.00	0.41
**Scenario 12 [MTD at Dose 1]**	Marginal prior	**0.64**	0.00	0.36
	Dose‐agnostic prior	0.61	0.00	0.39

*Note:* Bold indicates the calibration approach that allocates a greater mean proportion of patients to the best dose for each simulation scenario.

In Section S4 of the Supporting Information, we demonstrate that dose‐agnostic priors yield consistent reductions in PCS variability across other levels of dependence between C‐DLT and P‐DLT observations without sacrificing mean PCS. Regardless of dependency, we maintain scenario‐specific gains in PCS, with average PCS remaining robust under both strong and weak dependence scenarios.

With an inherent trade‐off between a priors willingness to explore the dose space and patient overdosing, trialists should assess the suitability of each approach for their specific trial. In Table S2 of the Supporting Information, we document this trade‐off in terms of the mean number of C‐DLTs and P‐DLTs observed during a trial. Across all 12 randomized scenarios, the number of observed C‐DLTs and P‐DLTs per trial is similar under marginally calibrated and dose‐agnostic priors. When dose‐agnostic priors are used, there is a maximum increase of 0.47 additional C‐DLTs and 0.52 additional P‐DLTs per trial relative to marginally calibrated priors.

## 4 Continual Reassessment Method for Efficacy and Clinician‐Reported Toxicity (Eff+Tox‐CRM)

The CRM design has also been extended to account for joint efficacy and toxicity endpoints including Wages and Tait's seamless adaptive design for molecularly targeted agents, which we identify as Eff+Tox‐CRM [23]. This model adapts the original CRM design to estimate both efficacy and toxicity using a power model, with an additional decision step to choose an efficacy skeleton $q=(q_{1},\ldots ,q_{K})$ to reflect the true dose‐efficacy relationship, which may be a unimodal or plateauing response pattern across dose. The goal of the Eff+Tox‐CRM is to identify the dose that maximizes efficacy while maintaining an acceptable toxicity rate.

For d∈𝒟, toxicity is estimated via the power model with parameter λ akin to the estimation of C‐DLT in Section 3, denoted as ${\hat{\pi }}_{C,d}$. We can estimate the probability of efficacy for each dose under all skeletons $q_{k}$ such that,

(19) $$\begin{array}{l} \pi_{E,d}=\mathbb{P}(Y_{E}=1|d)=q_{k,d}^{\exp(\zeta_{k})}\;\text{for}\;q_{k}\in \{1,\ldots ,K\}, \end{array}$$

where $q_{k,d}$ indicates the probability of response under skeleton $q_{k}$ at dose d.

The posterior probability that each efficacy skeleton is true is estimated using a Bayesian model selection approach with given prior probabilities $\tau =\{\tau (q_{1}),\ldots ,\tau (q_{K})\}$ for each skeleton. For $q_{k}^{*}$ the most probable efficacy skeleton, the efficacy estimate is given by,

(20) $$\begin{array}{l} {\hat{\pi }}_{E,d}=q_{k^{*},d}^{\exp({\hat{\zeta }}_{k^{*}})}, \end{array}$$

where ${\hat{\zeta }}_{k^{*}}$ is the a posterior mean point estimate for skeleton $q_{k}^{*}$.

For $𝒜=\{d:{\hat{\pi }}_{C,d}\leq p_{C}\}$ representing a set of admissible doses with respect to C‐DLT rates, interim dose recommendations within the trial firstly follow a randomization routine, after which doses are assigned to optimal dose recommendations using the following criterion,

(21) $$\begin{array}{l} d^{*}=\arg\underset{d\in {𝒜}_{n}}{\max}\;{\hat{\pi }}_{E,d}. \end{array}$$

Further methodological details for Eff+Tox‐CRM is provided in Section S5 of the Supporting Information. For this trial design, θ=(λ,q) are parameters defining the dose‐selection decision‐rule ν(θ), with associated hyperparameters $\phi =(\phi_{\lambda },\phi_{q})$. Similarly to the PRO‐CRM design, the decision rule for Eff+Tox‐CRM is,

(22) $$\begin{array}{l} \nu (\theta )=\min\{\nu_{C}(\lambda ),\nu_{E}(\text{q})\}, \end{array}$$

where

(23) $$\begin{array}{l} \nu_{C}(\lambda )=\arg\underset{d\in 𝒟}{\min}|v_{d}^{\hat{\lambda }}-p_{C}|\;\text{and}\;\nu_{E}(\text{q})=\arg\underset{d\in 𝒟}{\max}\;q_{k^{*},d}, \end{array}$$

and $p_{C}$ denotes target C‐DLT rate. If $\nu_{E}(\text{q})$ is larger than the $\nu_{C}(\lambda )$, then it is assumed that we are constrained by $\nu_{C}(\lambda )$ (which itself will have the optimal efficacy among admissible doses). Alternatively, if $\nu_{E}(\text{q})$ is smaller than the $\nu_{C}(\lambda )$ then $\nu_{E}(\text{q})$ is the best dose, with no additional efficacious benefit associated with larger doses.

### 4.1 Prior Calibration for Eff+Tox‐CRM

Existing calibration approaches for Eff+Tox‐CRM follow Lee and Cheung [9], with the prior distribution of λ and q marginally calibrated. Similarly to Section 3.1, to jointly calibrate parameters priors $\phi =(\phi_{\lambda },\phi_{q})$ we write $\mu_{\phi }(d)$ in terms of marginal recommendations $\nu_{C}(\lambda )$ and $\nu_{E}(\text{q})$ for dose‐selection decision‐making as defined by (22). For l denoting the most efficacious dose and F denoting the cumulative distribution functions for λ with hyperparameters $\phi_{\lambda }$,

(24) $$\begin{array}{l} \mu_{\phi }(d)=\left\{\begin{array}{ll} 1-(1-F(a_{d+1};\phi_{\lambda }))\mathbb{P}(l\geq d+1)\; & \text{if}\;d=1,\\ (1-F(a_{d};\phi_{\lambda }))\mathbb{P}(l\geq d)\; & \\ \;-\;(1-F(a_{d+1};\phi_{\lambda }))\mathbb{P}(l\geq d+1)\; & \text{if}\;1<d<M,\\ (1-F(a_{d};\phi_{\lambda }))\mathbb{P}(l=d)\; & \text{if}\;d=M, \end{array}\right. \end{array}$$

where ${\text{H}}_{\lambda }=\{[a_{1},a_{2}),\ldots ,(a_{M},a_{M+1}]\}$ similarly to (12).

Equation (24) summarizes the following scenarios:

(25) $$\begin{array}{ll} \mu_{\phi }(1) & =\left\{\begin{array}{ll} 1\; & \text{if}\;l=1,\\ \mathbb{P}(\nu_{C}(\lambda )=1)\; & \text{if}\;l>1, \end{array}\right.\\ \mu_{\phi }(d) & =\left\{\begin{array}{ll} 0\; & \text{if}\;l<d,\\ \mathbb{P}(\nu_{C}(\lambda )\geq d)\; & \text{if}\;l=d,\\ \mathbb{P}(\nu_{C}(\lambda )=M)\; & \text{if}\;l>d \end{array}\right.\\ \mu_{\phi }(M) & =\left\{\begin{array}{ll} 0\; & \text{if}\;l<M,\\ \mathbb{P}(\nu_{C}(\lambda )=M)\; & \text{if}\;l=M, \end{array}\right. \end{array}$$

Each skeleton q identifies unique doses as best. As such, to build a prior distribution associated with q, we construct a prior on the dose space ensuring $\sum_{d=1}^{M}\mathbb{P}(l=d)=1$. Accordingly, the probability mass function is formulated in terms of the Beta cumulative distribution function $\Psi (\cdot ,\alpha_{l},\beta_{l})$:

(26) $$\begin{array}{c} \mathbb{P}(l=d)=\left\{\begin{array}{ll} \Psi (C_{1}\;;\alpha_{l},\beta_{l}),\; & \text{if}\;d=1\\ \Psi (C_{d}\;;\alpha_{l},\beta_{l})-\Psi (C_{d-1}\;;\alpha_{l},\beta_{l}),\; & \text{if}\;2\leq d<M\\ 1-\Psi (C_{d-1}\;;\alpha_{l},\beta_{l}),\; & \text{if}\;d=M \end{array}\right., \end{array}$$

where $C_{j}=\frac{j}{M}\;\text{for}\;j=1,\ldots ,M-1.$

For model parameter q, we tune hyperparameters $\phi_{\text{q}}=(\alpha_{l},\beta_{l})$. Similarly to the PRO‐CRM, as $\mu_{\phi }(d)$ has an analytical form, suitable parameter priors can be found using (5) assessing the KL divergence between $\mu_{\phi }(d)$ and prior probability distribution P. The prior on ζ does not alter the a priori distribution on doses. As such we fix ζ∼HN(0,0.1) throughout this manuscript, with further discussion on the prior choice for ζ provided in Section 5 of the Supporting Information.

### 4.2 Eff+Tox‐CRM Simulation Set‐Up

Similarly to the PRO‐CRM simulation set‐up, we compare Eff+Tox‐CRM trials where priors are jointly and marginally calibrated to be dose‐agnostic in a simulation study. We consider an Eff+Tox‐CRM trial with 5 increasing doses and a sample size of 39 patients sequentially enrolled in cohorts of 3. DLTs and efficacy response are all observed before the next cohort of patients are enrolled. Target C‐DLT rate $p_{C}$ is 0.25. Table 4 shows the C‐DLT and efficacy skeletons ($q_{k},k\in \{1,\ldots ,9\}$) utilized in this simulation study.

**TABLE 4:** Skeleton for C‐DLT and efficacy probabilities as presented in Eff+Tox‐CRM [23].

d	1	2	3	4	5	d	1	2	3	4	5
$u_{d}$	0.02	0.10	0.25	0.44	0.62	$q_{5}$	0.60	0.50	0.40	0.30	0.20
$q_{1}$	0.20	0.30	0.40	0.50	0.60	$q_{6}$	0.60	0.60	0.60	0.60	0.60
$q_{2}$	0.30	0.40	0.50	0.60	0.50	$q_{7}$	0.50	0.60	0.60	0.60	0.60
$q_{3}$	0.40	0.50	0.60	0.50	0.40	$q_{8}$	0.40	0.50	0.60	0.60	0.60
$q_{4}$	0.50	0.60	0.50	0.40	0.30	$q_{9}$	0.30	0.40	0.50	0.60	0.60

#### 4.2.1 Marginally Calibrated Priors

Similarly to our simulation for PRO‐CRM, we utilize a gamma prior for parameter λ. Eff+Tox‐CRM is originally presented in a simulation study where λ and τ(k) are marginally dose‐agnostic. With these priors in mind, for the model to recognize larger doses as the best dose, not only must the toxicity model recognize larger doses as safe, but it must also recognize that larger doses have the largest probability of response. For example, for dose 5 to be recognized as the best dose, it must be identified as the MTD *and* most efficacious dose. This is challenging when priors for the toxicity model and efficacy skeletons are chosen to be independently dose‐agnostic–biasing a priori dose recommendations to smaller doses. This a priori bias is shown in Figure 4b.

**FIGURE 4:**
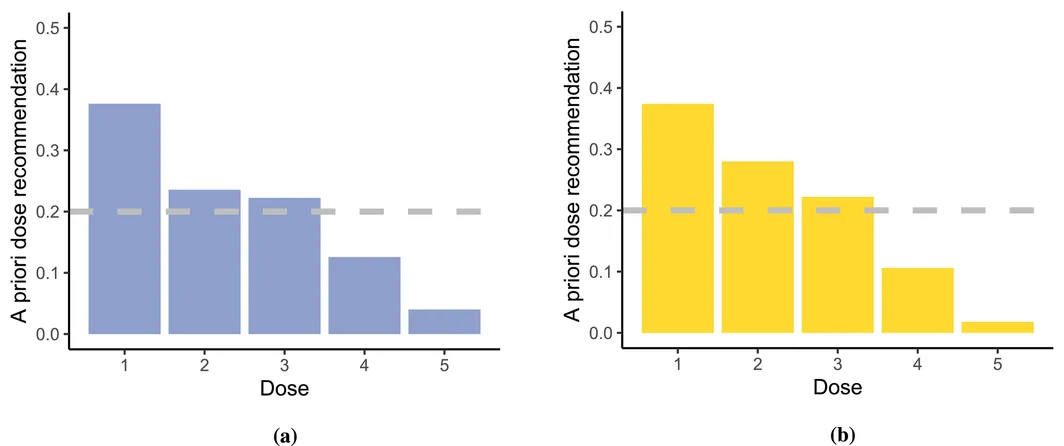
1,000 a priori dose recommendations for marginally calibrated dose‐agnostic prior recommendations for PRO‐CRM and Eff+Tox‐CRM trial design for 39 patients. The dotted line shows the respective dose‐agnostic recommendation for each trial design simulation scenario. (a) PRO‐CRM with $\lambda_{M}∼$ Gamma(2.78, 2.37) and $\xi_{M}∼$ Gamma(2.34, 1.96). (b) Eff+Tox‐CRM with $\lambda_{M}∼$ Gamma(2.78, 2.37) and $\tau_{M}(k)=\frac{1}{K}$.

Under a sample size of 39 with C‐DLT skeleton as per Table 4, priors calibrated marginally using existing dose‐agnostic prior methodology for a single outcome CRM are,

(27) $$\begin{array}{l} \lambda_{M}∼\text{Gamma}(2.78,2.37)\;\text{and}\;\tau_{M}(k)=\frac{1}{9}\forall k. \end{array}$$

#### 4.2.2 Dose‐Agnostic Priors

Similarly to Section 3.2, while priors can be calibrated for Eff+Tox‐CRM to suit any prior distribution, in this case study we jointly calibrate priors to be dose‐agnostic. For the Eff+Tox‐CRM, we wish to find hyperparameters $\phi =(\alpha_{\lambda },\beta_{\lambda },\alpha_{l},\beta_{l})$ for which their induced priors are dose‐agnostic. With infinitely many parameter solutions to calibrate jointly dose‐agnostic priors, we fix $\beta_{l}=1$ and evaluate design performance for varying values of $\alpha_{l}>1$, this ensures ℙ(l=j)≤ℙ(l=d)∀j<d (if one assumes an increasing dose‐toxicity relationship, one must also assume an increasing dose‐response relationship to calibrate dose‐agnostic priors). By setting $\alpha_{l}=\beta_{l}=1$ we return a marginally dose‐agnostic distribution on the skeletons. We allocate the prior mass for each dose equally among all skeletons that identify that dose as optimal.

We select the parameter values for dose‐agnostic priors when $\alpha_{l}=1.17$, supposing Clinician‐CRM recommendations are dose‐agnostic a priori and dose 5 is most likely to be most efficacious a priori, as such,

(28) $$\begin{array}{ll} \lambda_{J} & ∼\text{Gamma}(1.01,0.10)\;\text{and}\;\tau_{J}(k)\\ & =\{0.23,0.11,0.10,0.10,0.08,0.08,0.10,0.10,0.11\}. \end{array}$$

Figure 5 demonstrates the relationship between Clinician‐CRM recommendations and efficacy recommendations for varying $\alpha_{l}$ in this case study. This figure demonstrates the trade‐off between Clinician‐CRM and efficacy divergence for dose‐agnostic priors for Eff+Tox‐CRM. A simulation of a priori dose recommendations for these parameters is presented in Figure 2b.

**FIGURE 5:**
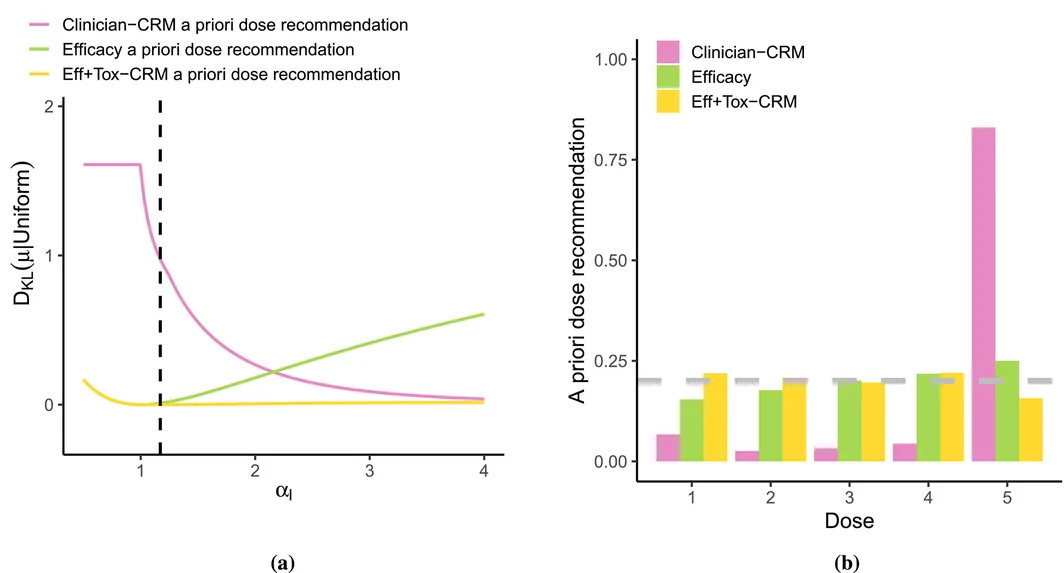
Comparison of a priori dose recommendations for Eff+Tox‐CRM, Clinician‐CRM and most efficacious dose. (a) KL divergence for a priori dose selections for Clinician‐CRM, efficacy and Eff+Tox‐CRM design with a dashed line indicating $\alpha_{l}=1.17.$ (b) 1,000 simulated a priori dose recommendations with $\alpha_{l}=1.17$ for Clinician‐CRM, efficacy and Eff+Tox‐CRM design.

#### 4.2.3 Hypothetical Trial Example for Eff+Tox‐CRM

In this section, we provide a hypothetical Phase I trial employing Eff+Tox‐CRM with marginally calibrated priors  (27) and dose‐agnostic priors  (28). The trial is simulated with 39 patients allocated sequentially in cohorts of 3, with probability of C‐DLT (0.01, 0.02, 0.05, 0.10, 0.20) and response (0.13, 0.25, 0.38, 0.50, 0.63). C‐DLT and efficacy response is simulated as per Cheung [30]. Under this scenario dose 5 is the best dose as it is both admissible from a safety perspective and has the best efficacy across all doses. To correctly identify this dose as best, the estimated DLT rate for dose 5 must be less than the maximum toxicity rate, requiring $\lambda \in H_{\lambda ,5}$ and selection of a skeleton recognizing dose 5 as best.

In Figure 6, we follow the allocation of patients to each dose. Figure 6b presents the cohorts for which λ lies in dose 5's H‐set. Figure 6c visually identifies which skeleton is identified as most likely to be true at each interim analysis. While dose‐agnostic priors recognize all doses admissible throughout the trial, an Eff+Tox‐CRM utilizing marginally calibrated priors only recognizes dose 5 as safe following the enrollment of the sixth cohort of patients. Hence after Cohort 5 and for the remainder of the trial, dose allocation is solely determined by observed efficacy responses. Marginally calibrated dose‐agnostic priors are risk‐averse and never explore the largest dose which is also the best dose. However, from Cohort 4 onwards, the Eff+Tox‐CRM design utilizing jointly calibrated dose‐agnostic priors consistently recognizes correctly that dose 5 is the best dose until the end of trial and at final analysis. The Eff+Tox‐CRM design which utilizes marginally calibrated priors incorrectly identifies the smaller dose, dose 4 as the best dose at the end of the trial.

**FIGURE 6:**
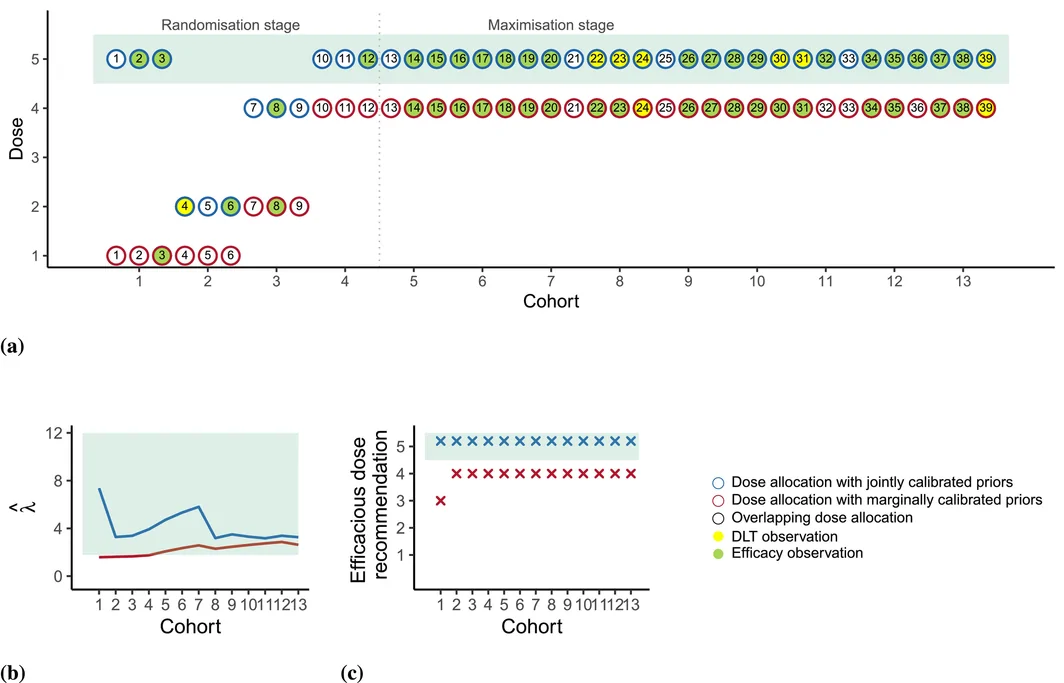
Hypothetical dose‐finding trial using Eff+Tox‐CRM trial design with true C‐DLT rates (0.01, 0.02, 0.05, 0.10, 0.20) and true response rates (0.13, 0.25, 0.38, 0.50, 0.63) for priors calibrated as per (27) shown in red and the trial with the dose‐agnostic priors is shown in blue (28). A total of 39 patients are treated in cohorts of 3. (a) Recommended doses at each interim analysis. Dose 5, marked in green is the best dose. Patients 24 and 39 experience both a C‐DLT and response. The dotted line indicates the end of run‐in, where patients were allocated to dose using an adaptive randomization routine. (b) C‐DLT exponent estimate at each interim analysis with the H‐set for the MTD (dose 5) highlighted in green. (c) Most efficacious dose as identified at each interim analyzes with optimal dose (dose 5) highlighted in green.

#### 4.2.4 Ensemble Simulation Study

As per Section 3.2.2, we demonstrate the performance of dose‐agnostic priors for Eff+Tox‐CRM within an ensemble simulation study [31], supporting the evaluation of Eff+Tox‐CRM across all possible configurations of dose‐toxicity and dose‐efficacy relationships one may expect to see in a trial. Under this trial design, six clinician‐MTD configurations are possible (similarly to Section 3.2.2) and nine dose‐efficacy relationships are possible (see Table 4). As such, we investigate 54 possible simulation scenarios, with 5000 trials simulated for each scenario. In Section S6 of the Supporting Information, we document all 54 investigated simulation scenarios. To evaluate the performance of dose‐agnostic priors, we examine the standard deviation of the PCS and mean PCS across all 54 scenarios. We further present twelve randomly selected scenarios from the full set of 54 to illustrate the impact of different prior choices on scenario‐specific PCS. Simulation results of all 54 scenarios can be found at https://github.com/alemily100/calibrate_priors.

### 4.3 Results

Across all 54 scenarios, when one dose is best, utilizing Eff+Tox‐CRM with jointly calibrated dose‐agnostic priors compared to marginally calibrated priors reduces the standard deviation of PCS while maintaining mean PCS performance. Specifically, the standard deviation of PCS when one dose is best for the Eff+Tox‐CRM design is reduced from 0.17 under marginally calibrated priors to 0.13 under dose‐agnostic priors. This demonstrates that utilizing dose‐agnostic priors can strengthen the robustness of PCS across all potential MTD scenarios compared to existing marginal calibration approaches. Across all scenarios, dose‐agnostic priors maintain a mean PCS of 0.45, very similar to the mean PCS under marginally calibrated priors of 0.44.

Table 5 presents 12 randomly selected scenarios for each prior specification, illustrating the advantages of dose‐agnostic priors in scenario‐specific settings. The robustness of the dose‐agnostic prior choice is most evident in Scenario D where the largest dose (dose 5) is best. In this scenario, an Eff+Tox‐CRM trial utilizing dose‐agnostic priors correctly recognizes dose 5 as best 24% of the time–improving upon PCS for an Eff+Tox‐CRM using marginally calibrated priors by approximately 20%. This performance does not forsake good performance in other simulation scenarios, with dose‐agnostic priors performing approximately as well or better than marginally calibrated priors in each presented scenario.

**TABLE 5:** Proportion of MTD selection recommendations for each dose under 12 random scenarios for Eff+Tox‐CRM for marginally calibrated (27) and dose‐agnostic priors (28). The optimal dose under each scenario is presented in green. Best dose(s) sums all doses which are admissible with optimal efficacy rates.

		Dose level					
		1	2	3	4	5	Best dose(s)
**Scenario A**	Probability of Clinician‐DLT	0.05	0.10	0.12	0.15	0.20	
	Probability of efficacy	0.63	0.63	0.63	0.63	0.63	
	Marginal recommendation	0.56	0.26	0.14	0.03	0.00	1.00
	Dose‐agnostic recommendation	0.26	0.34	0.23	0.07	0.08	0.98
**Scenario B**	Probability of Clinician‐DLT	0.05	0.10	0.12	0.15	0.20	
	Probability of efficacy	0.50	0.63	0.50	0.38	0.25	
	Marginal recommendation	0.27	0.54	0.15	0.03	0.00	0.54
	Dose‐agnostic recommendation	0.14	0.57	0.19	0.06	0.02	0.57
**Scenario C**	Probability of Clinician‐DLT	0.10	0.12	0.15	0.20	0.25	
	Probability of efficacy	0.38	0.50	0.63	0.63	0.63	
	Marginal recommendation	0.09	0.37	0.44	0.07	0.00	0.51
	Dose‐agnostic recommendation	0.04	0.26	0.45	0.12	0.08	0.65
**Scenario D**	Probability of Clinician‐DLT	0.10	0.12	0.15	0.20	0.25	
	Probability of efficacy	0.13	0.25	0.38	0.50	0.63	
	Marginal recommendation	0.02	0.17	0.45	0.30	0.05	0.05
	Dose‐agnostic recommendation	0.01	0.09	0.29	0.33	0.24	0.24
**Scenario E**	Probability of Clinician‐DLT	0.12	0.15	0.20	0.25	0.40	
	Probability of efficacy	0.50	0.63	0.63	0.63	0.63	
	Marginal recommendation	0.30	0.51	0.14	0.01	0.00	0.66
	Dose‐agnostic recommendation	0.14	0.49	0.23	0.06	0.01	0.79
**Scenario F**	Probability of Clinician‐DLT	0.12	0.15	0.20	0.25	0.40	
	Probability of efficacy	0.38	0.50	0.63	0.63	0.63	
	Marginal recommendation	0.12	0.44	0.35	0.03	0.00	0.39
	Dose‐agnostic recommendation	0.07	0.36	0.40	0.08	0.01	0.48
**Scenario G**	Probability of Clinician‐DLT	0.15	0.20	0.25	0.40	0.55	
	Probability of efficacy	0.38	0.50	0.63	0.50	0.38	
	Marginal recommendation	0.22	0.48	0.19	0.00	0.00	0.19
	Dose‐agnostic recommendation	0.15	0.43	0.25	0.01	0.00	0.25
**Scenario H**	Probability of Clinician‐DLT	0.15	0.20	0.25	0.40	0.55	
	Probability of efficacy	0.25	0.38	0.50	0.63	0.63	
	Marginal recommendation	0.17	0.44	0.28	0.01	0.00	0.28
	Dose‐agnostic recommendation	0.13	0.37	0.32	0.02	0.00	0.32
**Scenario I**	Probability of Clinician‐DLT	0.20	0.25	0.40	0.55	0.60	
	Probability of efficacy	0.50	0.63	0.63	0.63	0.63	
	Marginal recommendation	0.42	0.28	0.01	0.00	0.00	0.28
	Dose‐agnostic recommendation	0.28	0.32	0.01	0.00	0.00	0.32
**Scenario J**	Probability of Clinician‐DLT	0.20	0.25	0.40	0.55	0.60	
	Probability of efficacy	0.38	0.50	0.63	0.50	0.38	
	Marginal recommendation	0.35	0.33	0.03	0.00	0.00	0.33
	Dose‐agnostic recommendation	0.26	0.34	0.03	0.00	0.00	0.34
**Scenario K**	Probability of Clinician‐DLT	0.20	0.25	0.40	0.55	0.60	
	Probability of efficacy	0.25	0.38	0.50	0.63	0.63	
	Marginal recommendation	0.30	0.38	0.03	0.00	0.00	0.38
	Dose‐agnostic recommendation	0.24	0.35	0.05	0.00	0.00	0.35
**Scenario L**	Probability of Clinician‐DLT	0.20	0.25	0.40	0.55	0.60	
	Probability of efficacy	0.13	0.25	0.38	0.50	0.63	
	Marginal recommendation	0.28	0.39	0.04	0.00	0.00	0.39
	Dose‐agnostic recommendation	0.22	0.34	0.05	0.00	0.00	0.34

In Table 6 we detail the mean proportion of patients assigned to the best dose(s), underdosed (with suboptimal efficacy) and overdosed (with inadmissible C‐DLT rate) for these 54 scenarios. Across these scenarios, the proportion of patients assigned to a best dose is always higher when dose‐agnostic priors are utilized rather than marginally calibrated priors, particularly in Scenario D where 19% more patients assigned to the best dose when dose‐agnostic priors are used. However this improvement is traded off with more patients overdosed, most notably in Scenarios B, I‐L where approximately 7%–11% more patients are allocated to doses larger than the best dose compared to a more risk‐averse trial which utilizes marginally calibrated priors.

**TABLE 6:** Proportion of patients correctly dosed, underdosed or overdosed for each scenario for Eff+Tox‐CRM design, for marginally calibrated and dose‐agnostic priors.

		Best dose	Underdosed	Overdosed
**Across all ensemble scenarios**	Marginal prior	**0.53**	0.37	0.10
	Dose‐agnostic prior	0.47	0.30	0.23
**Scenario A [Doses 1‐5 best doses]**	Marginal prior	**1.00**	0.00	0.00
	Dose‐agnostic prior	**1.00**	0.00	0.00
**Scenario B [Dose 2 best dose]**	Marginal prior	0.41	0.33	0.26
	Dose‐agnostic prior	**0.44**	0.19	0.37
**Scenario C [Doses 3‐5 best dose]**	Marginal prior	0.42	0.58	0.00
	Dose‐agnostic prior	**0.57**	0.43	0.00
**Scenario D [Dose 5 best dose]**	Marginal prior	0.03	0.97	0.00
	Dose‐agnostic prior	**0.22**	0.78	0.00
**Scenario E [Doses 2‐4 best doses]**	Marginal prior	0.61	0.39	0.00
	Dose‐agnostic prior	**0.74**	0.23	0.04
**Scenario F [Doses 3‐4 best doses]**	Marginal prior	0.35	0.65	0.00
	Dose‐agnostic prior	**0.43**	0.53	0.05
**Scenario G [Dose 3 best dose]**	Marginal prior	0.22	0.76	0.02
	Dose‐agnostic prior	**0.28**	0.65	0.06
**Scenario H [Dose 3 best dose]**	Marginal prior	0.28	0.68	0.04
	Dose‐agnostic prior	**0.32**	0.59	0.10
**Scenario I [Dose 2 best dose]**	Marginal prior	0.31	0.55	0.14
	Dose‐agnostic prior	**0.39**	0.42	0.20
**Scenario J [Dose 2 best dose]**	Marginal prior	0.36	0.49	0.15
	Dose‐agnostic prior	**0.40**	0.38	0.22
**Scenario K [Dose 2 best dose]**	Marginal prior	**0.39**	0.43	0.18
	Dose‐agnostic prior	**0.39**	0.35	0.26
**Scenario L [Dose 2 best dose]**	Marginal prior	**0.40**	0.41	0.19
	Dose‐agnostic prior	0.39	0.35	0.27

*Note:* Bold indicates the calibration approach that allocates a greater mean proportion of patients to the best dose for each simulation scenario.

In Table S5 of the Supporting Information, we illustrate the mean number of C‐DLTs and efficacy responses observed for each simulation scenario when priors are marginally or jointly calibrated to be dose‐agnostic. The trade‐off between observed C‐DLTs and efficacy responses under each prior mirrors our intuition that dose‐agnostic priors promote the exploration of all doses while marginally calibrated priors are more risk‐averse and prefer exploring lower doses.

For the joint calibration of dose‐agnostic priors the Eff+Tox‐CRM trial, in Section S7 of the Supporting Information we investigate the role of $\alpha_{l}$ on trial operating characteristics. When $\alpha_{l}\in \{1.03,1.17,1.88,2.51,3.89\}$ we show that while priors maintain approximately equal standard deviation of PCS and mean PCS, scenario‐specific performance may differ with larger choices of $\alpha_{l}$ associated with more patient overdosing.

## 5 General Guidance for Calibrating Joint Outcome Dose‐Finding Designs

In this section, we provide further general recommendations to support trialists calibrating their model‐based joint outcome dose‐finding trial designs.

### 5.1 Accuracy and Efficiency of Analytical Prior Calibration for Joint Outcome Trial Designs

Dose‐agnostic priors are just one example of a prior which trialists may wish to use for their joint outcome trial design. As well as calibrating dose‐agnostic priors, the analytical calibration of priors presented in Section 2 can be utilized to calibrate any prior deemed appropriate by a trial team. In this section, we demonstrate the advantages of this analytical prior calibration over a traditional grid search for calibrating elicited priors in terms of both accuracy and computational efficiency. Figure 7 demonstrates the number of times the KL divergence must be evaluated to identify priors for the PRO‐CRM using either our analytical joint calibration approach or a traditional grid search for four potential priors. Similarly to the proposed joint calibration approach, the grid search sought the hyperparameters that minimized the KL divergence between the induced parameter prior and each target prior in turn. We evaluated the grid search over ${𝒢}_{n}^{4}$, where ${𝒢}_{n}$ is a n‐point equally spaced grid on [0.01, 10] for each of four parameters ($\alpha_{\lambda },\beta_{\lambda },\alpha_{\xi },\beta_{\xi }$) for n=2,…,10. For n=1 the midpoint was used. For each n, this yielded $n^{4}$ candidate hyperparameter combinations for which the empirical KL divergence was evaluated using 1000 sampled parameter priors. The hyperparameter combination with the smallest empirical KL divergence was then selected.

**FIGURE 7:**
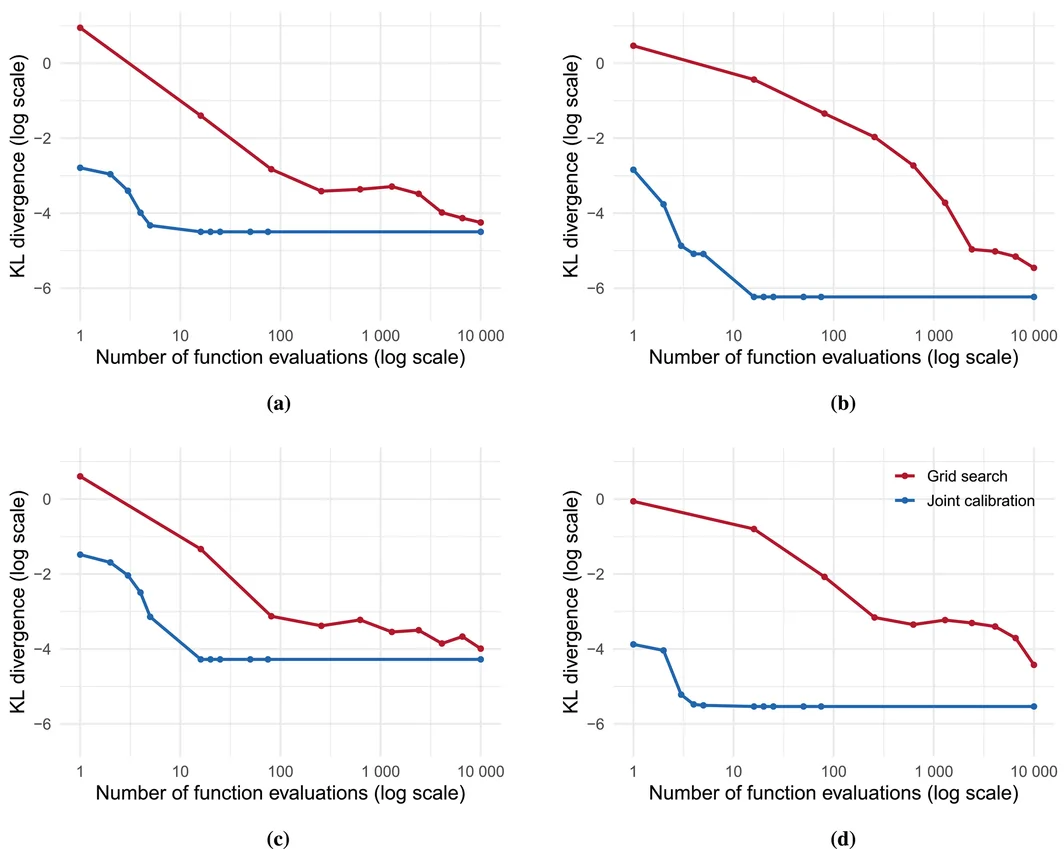
The number of evaluations of the KL divergence (function evaluations) required to minimize the KL divergence between the true elicited prior and prior computed for the PRO‐CRM using a grid search or joint calibration technique presented in Section 2. (a) KL divergence to the true prior (0.35, 0.30, 0.20, 0.10, 0.05), (b) KL divergence to the true prior (0.10, 0.15, 0.20, 0.25, 0.30), (c) KL divergence to the true prior (0.10, 0.20, 0.40, 0.20, 0.10), (d) KL divergence to the true prior (0.20, 0.20, 0.20, 0.20, 0.20).

Across all four scenarios, the joint calibration approach converges to parameter values that minimize the KL divergence between the true and computed priors within approximately 16 iterations. In contrast, a grid search over $10^{4}$ possible combinations with 10,000 function evaluations still yields parameter values with a higher KL divergence than those obtained via joint calibration. This efficiency is also reflected in the computational time. Figure 7a demonstrates the number of function evaluations required to calibrate the prior (0.35, 0.30, 0.20, 0.10, 0.05) for the PRO‐CRM trial. Using the statistical software R, the joint calibration approach identifies a prior with the smallest KL divergence in 0.01 secs, whereas a traditional grid search requires 38 s and selects a prior with a larger KL divergence.

### 5.2 Skeleton Calibration Guidance Joint Outcome CRM Trials

In this section, we present a simulation‐based strategy to calibrate skeletons for CRM‐based joint outcome dose‐finding trial designs. For the single outcome CRM, previous work has shown that to achieve good CRM performance, a trialist may either (i) fix the skeleton and calibrate the prior or (ii) fix the prior and calibrate the skeleton [9]. As such, this paper focuses on the calibration of model‐based joint outcome trial designs by tuning parameter priors with fixed skeletons. As optimal design performance depends on the interplay between the skeleton and the calibration of parameter priors [32], we recommend that trialists wishing to calibrate both components evaluate the performance of alternative skeletons through simulation studies, with priors derived from each skeleton using the methodology presented in this paper. This ensures that parameter priors are consistent with trialist's a priori beliefs while ensuring the joint outcome designs' optimal performance.

As per Lee and Cheung [28], CRM skeletons can be calibrated using indifference intervals δ which identify a range of acceptable toxicity probabilities and provides a systematic approach for selecting skeletons. Trialists wishing to evaluate the performance of their CRM joint outcome trial design with different skeletons may complete ensemble simulations (as implemented in Sections 3.2.2 and 4.2.2) where skeletons are calibrated with different indifference intervals δ and parameter priors are tuned for each skeleton accordingly. We provide one example case study of this approach for the Eff+Tox‐CRM design in Section S8 of the Supporting Information with skeletons calibrated using the getprior() function in package dfcrm implemented in R [29].

### 5.3 Prior Calibration When $\mu_{\phi }$ Is Not Available in Closed Form

For both the PRO‐CRM and Eff+Tox‐CRM, $\mu_{\phi }$ can be calculated analytically which enables efficient prior calibration. More generally, our framework can be extended to calibrate priors on the dose space for trial designs such as EffTox [33] which use more complex utility‐based decision criteria for which $\mu_{\phi }$ cannot be written in closed form. In Section S9 of the Supporting Information, we demonstrate how the framework presented in this paper can be used to calibrate priors for EffTox when $\mu_{\phi }$ is estimated empirically via Monte Carlo simulation.

## 6 Discussion

This paper emphasizes the critical role of effective prior calibration in enabling the essential joint outcome driven decision‐making for robust dose selection in contemporary dose‐finding trials. We present a general methodological framework for the calibration of priors for joint outcome model‐based dose‐finding trial designs. The joint calibration of dose‐agnostic priors for two case studies demonstrates the important role of priors in the effective exploration of dose spaces within trials. This may be particularly beneficial in instances where larger doses are truly best but may be under explored using existing, risk‐averse marginal calibration techniques.

To reflect popular prior choices within single outcome CRM designs, we present two case studies demonstrating the role of priors on model performance when priors are calibrated to be dose‐agnostic. The jointly calibrated dose‐agnostic priors utilized within this paper demonstrate robustness to simulation scenario by reducing the standard deviation of PCS across all ensemble simulations while maintaining mean PCS. This illustrates how dose‐agnostic priors can yield less variability in PCS when knowledge on which dose is best is not known a priori. However, while both the PRO‐CRM and Eff+Tox‐CRM designs demonstrate the benefits of dose‐agnostic priors, utilizing such priors can be associated with a greater proportion of patients overdosed compared to existing calibration techniques which become risk‐averse when utilized for joint outcome trial designs.

To support uptake in practice, the methodology presented for the calibration of priors is kept simple. However future work could evaluate the performance of priors with the addition of copulas to account for dependence between trial endpoints involved in dose‐selection decision‐making. Such priors may reduce the proportion of patients overdosed within a trial, though copula models may not necessarily improve upon PCS in comparison to independent likelihood models [34].

Within this paper, we specify priors dependent on the interpretable a priori dose‐selection outcome. This allows trialists to explore parameter priors with reference to the real‐world outcomes they influence (the probability of a priori dose selection), facilitating prior elicitation and providing insight into how different prior selections affect dose decision‐making. Our framework provides a computationally efficient means of translating a trialist's pretrial quantitative beliefs into corresponding priors on the design parameters. When compared to a traditional grid search, the analytical methodological framework presented in this paper can select suitable priors up to 4000 times faster and with improved accuracy.

While the KL divergence is a commonly used and established measure for parameter calibration in Bayesian dose‐finding trials [5], practitioners may also wish to consider other statistical divergences they deem relevant. Building on existing research demonstrates that good CRM performance can be achieved by fixing either the prior parameters or the skeleton while calibrating the other [9], this work focuses on the calibration of joint outcome CRM trials through the calibration of prior parameters. We explore the performance of the Eff+Tox‐CRM under this routine when skeletons are calibrated as per existing marginal skeleton calibration methodology in the Supporting Information. An important direction for future research is the development of principled analytical approaches for the calibration of skeletons in joint outcome CRM designs, which may further improve operating characteristics for such designs.

We hope this paper not only serves as a resource for trialists wishing to calibrate priors for joint outcome trials, but also highlights the broader values of thoughtful prior calibration in guiding trial decision‐making and improving trial design performance. Harnessing the power of well‐calibrated priors can support the incorporation of clinical expertise within trials and ensure superior trial outcomes. As Bayesian methods continue to evolve, recognizing the importance of priors and their careful calibration will be essential for advancing multi‐outcome decision‐making, which is expected to play an increasingly significant role in DFOT designs in the years ahead.

## Author Contributions

Emily Alger: conceptualization, methodology, software, formal analysis, investigation, data curation, writing – original draft, writing – review and editing, visualization, project administration. Shing M. Lee: methodology, writing – review and editing, supervision. YKC: methodology, writing – review and editing, supervision. Christina Yap: methodology, writing – review and editing, supervision, project administration, funding acquisition.

## Funding

EA has been supported to undertake this work as part of a PhD studentship from the Institute of Cancer Research within the MRC/NIHR Trials Methodology Research Partnership.

## Conflicts of Interest

The authors declare no conflicts of interest.

## Supporting information




**Table S1:** Ensemble simulation scenarios for PRO‐CRM.
**Table S2:** Mean number of C‐DLTs and P‐DLTs per PRO‐CRM trial for 1,250 simulations for marginally calibrated and doseagnostic priors.
**Table S3:** Proportion of MTD selection recommendations for each dose under 12 random scenarios for PRO‐CRM with marginally and jointly calibrated priors with C‐DLT and P‐DLT correlation parameters φ ∈ {0.1, 3.0}.
**Figure S1:** Hypothetical dose‐finding trial using Eff+Tox‐CRM with DLT rates (0.05, 0.1, 0.2, 0.28, 0.5) and efficacy rates (0.6, 0.6, 0.6, 0.6, 0.6). A total of 39 patients are treated in cohorts of 3.
**Figure S2:** Hypothetical dose‐finding trial using Eff+Tox‐CRM with DLT rates (0.05, 0.1, 0.2, 0.28, 0.5) and efficacy rates (0.6, 0.6, 0.6, 0.6, 0.6). A total of 39 patients are treated in cohorts of 3.
**Table S4:** Ensemble simulation scenarios for Eff+Tox‐CRM.
**Table S5:** Mean number of C‐DLTs and P‐DLTs per Eff+Tox‐CRM trial for 1,250 simulations for marginally calibrated and dose‐agnostic priors.
**Figure S3:** Mean proportion of patients treated at the best dose(s), underdosed and overdosed across 5,000 simulations for 12 scenarios when priors are jointly calibrated for different choices of α_l_.
**Table S6:** Skeleton for C‐DLT and efficacy probabilities as using getpriori() function in dfcrm package in R and δ_C_ = 0.05.
**Figure S4:** Proportion of best dose(s) selection recommendations for each dose under 12 scenarios using getprior() function in dfcrm with three different half‐widths with priors which are calibrated as per the modified marginal and jointly calibrated approach.
**Figure S5:** A priori dose recommendations under four potential prior‐calibration scenarios for the EffTox[1] trial design, with the true scenario shown in black and the calibrated empirical prior recommendation shown in green.

## Data Availability

The data that support the findings of this study are openly available in calibrate_priors at https://github.com/alemily100/calibrate_priors.
