# Supplementary material for: Calibration of Priors for Bayesian Model‐Based Dose‐Finding Trial Designs With Joint Outcomes
Source: Stat Med. 2026 Oct 1;45(23-24):e70746. doi: 10.1002/sim.70746 (PMC13628446; doi:10.1002/sim.70746)
Supplement: Supplementary file 1 [file SIM-45-0-s001.pdf]

SUPPLEMENTARY MATERIALS

Calibration of priors for Bayesian model-based dose-finding trial designs with joint outcomes

1 | SUPPLEMENTARY DETAILS FOR SECTION 2.2.1

In this section we provide further details of results presented in Section 2.2.1 of the main manuscript to demonstrate that dose-agnostic priors calibrated marginally are not dose-agnostic jointly. Consider a trial with M doses, with marginal recommendations d_1^* and d_2^* such that,

$$\mathbb{P}(d_1^* = i) = \frac{1}{M} \; \forall \; i \in \{1, ..., M\} \tag{1}$$

$$\mathbb{P}(d_2^* = j) = \frac{1}{M} \; \forall \; j \in \{1, ..., M\}. \tag{2}$$

In this case,

$$\begin{aligned}
\mathbb{P}(\min(d_1^*, d_2^*) = d) &= \mathbb{P}(\min(d_1^*, d_2^*) \geq d) - \mathbb{P}(\min(d_1^*, d_2^*) \geq d + 1), &(3)\\
&= \mathbb{P}(d_1^* \geq d, d_2^* \geq d) - \mathbb{P}(d_1^* \geq d + 1, d_2^* \geq d + 1), &(4)\\
&= \mathbb{P}(d_1^* \geq d)\mathbb{P}(d_2^* \geq d) - \mathbb{P}(d_1^* \geq d + 1)\mathbb{P}(d_2^* \geq d + 1), &(5)\\
&= \left(\frac{M - d + 1}{M}\right)^2 - \left(\frac{M - d}{M}\right)^2, &(6)\\
&= \frac{2(M - d) + 1}{M^2}. &(7)
\end{aligned}$$

To identify a dose d for which the probability mass is greater under jointly calibrated dose-agnostic priors than under marginally calibrated dose-agnostic priors, we wish to find d such that,

$$\frac{2(M - d) + 1}{M^2} \leq \frac{1}{M}. \tag{8}$$

Accordingly,

$$\begin{aligned}
2(M - d) + 1 &\leq M, &(9)\\
2d &\geq M + 1, &(10)\\
d &\geq \frac{M + 1}{2}. &(11)
\end{aligned}$$

2 | PRO-CRM ADDITIONAL DETAILS

In this section we provide further methodological details of the PRO-CRM design. Given data available for n patients on the trial D_n, the likelihood of observing C-DLTs is based on a power model such that,

$$L_n(\lambda | D_n) = \prod_{i=1}^{n} \{1 - v_{(i)}^{\lambda}\}^{1(Y_{C,i}=0)} \{v_{(i)}^{\lambda}\}^{1(Y_{C,i}=1)}, \tag{12}$$

where $v_{(i)}$ indicates the prior C-DLT probability associated with patient i's dosage.
With $\text{supp}(\lambda) = \Lambda$ and prior distribution $P(\lambda)$ placed on λ, Bayesian point estimate $\hat{\lambda}$ is estimated as,

$$\hat{\lambda} = \int_{\Lambda} \lambda P(\lambda | D_n) d\lambda \text{, where } P(\lambda | D_n) = \frac{L_n(\lambda | D_n) P(\lambda)}{\int_{\Lambda} L_n(\lambda | D_n) P(\lambda) d\lambda}. \tag{13}$$

Point estimate $\hat{\xi}$ can be estimated accordingly when the likelihood of P-DLT is constructed similarly to (12).

3 | PRO-CRM ENSEMBLE SIMULATION SCENARIOS

In Table S1 we present the ensemble simulation scenarios investigated in the PRO-CRM simulation study. We investigate 6 C-DLT rate scenarios and 6 P-DLT scenarios, with 36 (6×6) scenarios investigated in total.

C-DLT rate ensemble scenarios					P-DLT rate ensemble scenarios				
Dose 1	Dose 2	Dose 3	Dose 4	Dose 5	Dose 1	Dose 2	Dose 3	Dose 4	Dose 5
0.05	0.10	0.12	0.15	0.20	0.04	0.09	0.17	0.20	0.25
0.10	0.12	0.15	0.20	0.25	0.09	0.17	0.20	0.25	0.35
0.12	0.15	0.20	0.25	0.40	0.17	0.20	0.25	0.35	0.50
0.15	0.20	0.25	0.40	0.55	0.20	0.25	0.35	0.50	0.65
0.20	0.25	0.40	0.55	0.60	0.25	0.35	0.50	0.65	0.80
0.25	0.40	0.55	0.60	0.70	0.35	0.50	0.65	0.80	0.85

Table S1 Ensemble simulation scenarios for PRO-CRM.

4 | SUPPLEMENTARY PRO-CRM SIMULATION RESULTS

This section provides further simulation studies beyond those presented in the main manuscript for PRO-CRM. In Table S2 we present the mean number of DLTs observed per trial across all 1,250 simulation runs with 12 random simulation scenarios in instances where priors are marginally calibrated or jointly calibrated to be dose-agnostic. Whilst utilising dose-agnostic priors increases the mean number of C-DLTs and P-DLTs observed on a PRO-CRM trial, compared to an instance where marginal priors are utilised, this increase is no more than 0.52 additional C-DLT or P-DLTs.

Whilst the main manuscript evaluates the performance of PRO-CRM when the correlation between C-DLT and P-DLT rate is set to $\varphi = 0.9$ (with endpoints moderately correlated), in Table S3 we evaluate the performance of marginally calibrated and dose-agnostic priors when C-DLT and P-DLT are very strongly correlated ($\varphi = 0.1$) and very weakly correlated ($\varphi = 3.0$).

Irrespective of the correlation between C-DLT and P-DLT rate, dose-agnostic priors yield a consistent improvement in the standard deviation of PCS across all 36 ensemble scenarios considered, reducing the standard deviation of PCS from 0.15 and 0.18 when priors are calibrated marginally to 0.12 and 0.15 when priors are calibrated jointly and $\varphi = 0.1$ and $\varphi = 3.0$ respectively. Importantly, this reduction in standard deviation does not come at the expense of mean PCS, which remains steady at 0.60 when priors are both marginally calibrated and jointly calibrated to be dose-agnostic. What's more, the scenario-specific improvement in PCS performance for priors calibrated using dose-agnostic priors (particularly in Scenario 1) is robust to the observed correlation between C-DLT and P-DLT.

	Mean number of C-DLTs per trial		Mean number of P-DLTs per trial	
	Marginal	Dose-agnostic	Marginal	Dose-agnostic
**Scenario 1 [MTD at Dose 5]**	5.38	5.69	6.91	7.31
**Scenario 2 [MTD at Dose 3]**	4.17	4.35	12.53	13.05
**Scenario 3 [MTD at Dose 2]**	4.66	4.99	14.35	14.60
**Scenario 4 [MTD at Dose 4]**	7.75	8.11	7.74	8.02
**Scenario 5 [MTD at Dose 1]**	5.44	5.32	16.27	16.48
**Scenario 6 [MTD at Dose 3]**	9.48	9.87	5.21	5.40
**Scenario 7 [MTD at Dose 3]**	8.89	9.36	8.84	9.18
**Scenario 8 [MTD at Dose 2]**	7.83	7.95	13.80	14.11
**Scenario 9 [MTD at Dose 2]**	11.02	11.25	6.14	6.30
**Scenario 10 [MTD at Dose 2]**	10.76	11.02	8.18	8.23
**Scenario 11 [MTD at Dose 1]**	12.33	12.53	8.76	8.72
**Scenario 12 [MTD at Dose 1]**	12.13	12.31	11.39	11.52

Table S2 Mean number of C-DLTs and P-DLTs per PRO-CRM trial for 1,250 simulations for marginally calibrated and dose-agnostic priors.

5 | EFF+TOX-CRM ADDITIONAL DETAILS

5.1 | Design details

In this section we provide further methodological details on the Eff+Tox-CRM design. Given data available for n patients on the trial D_n, the likelihood of observing an efficacy response is based on a power model such that,

$$L_{n,k}(\zeta|D_n) = \prod_{i=1}^{N} \{1 - q_{k,(i)}^{\exp(\zeta)}\}^{1(Y_{E,i}=0)} \{q_{k,(i)}^{\exp(\zeta)}\}^{1(Y_{E,i}=1)}, \tag{14}$$

where $q_{k,(i)}$ indicates the prior efficacy probability associated with patient i's dosage for skeleton q_k. With $\text{supp}(\zeta) = Z$ and prior distribution $h(\zeta)$, we now wish to identify which skeleton q_k best reflects the observed data. We may estimate the posterior probability of each efficacy skeleton given prior probabilities $\boldsymbol{\tau} = \{\tau(1), ..., \tau(K)\}$ such that,

$$w(k|D_n) = \frac{\tau(k)\int_Z L_{n,k}(\zeta|D_n)h(\zeta)d\zeta}{\sum_{k=1}^{K} \tau(k)\int_Z L_{n,k}(\zeta|D_n)h(\zeta)d\zeta}. \tag{15}$$

For the skeleton $k^* = \arg\max_{k\in\{1,...,K\}} w(k|D_n)$ which best reflects the observed data, and $\text{supp}(\zeta) = Z$ the Bayesian point estimate $\hat{\zeta}_{k^*}$ is estimated as,

$$\hat{\zeta}_{k^*} = \int_Z \zeta P_{k^*}(\zeta|D_n)d\zeta \;\text{ with }\; P_{k^*}(\zeta|D_n) = \frac{L_{n,k^*}(\zeta|D_n)h(\zeta)}{\int_Z L_{n,k^*}(\zeta|D_n)h(\zeta)d\zeta}. \tag{16}$$

As such, we estimate the efficacy rates for each dose in the trial $\hat{\pi}_{E,d}$ such that,

$$\hat{\pi}_{E,d} = q_{k^*,d}^{\exp\hat{\zeta}_{k^*}} \tag{17}$$

We now discuss the adaptive dose-escalation routine undertaken by Eff+Tox-CRM in the trial.

Commencement of trial

At the commencement of the trial, the starting dose is selected as the admissible dose (based on the elicited clinician skeleton) with the highest probability of response according to the prior probability assigned to each skeleton, $\tau(k)$. For scenarios with equally likely a priori dose-response relationships, the chosen skeleton is randomly selected among these.

			Dose level				
			1	2	3	4	5
**Scenario 1**	**Marginal recommendation**	$\varphi = 0.1$	0.00	0.01	0.09	0.40	0.50
		$\varphi = 3.0$	0.00	0.01	0.10	0.47	0.42
	**Dose-agnostic recommendation**	$\varphi = 0.1$	0.00	0.00	0.06	0.30	0.63
		$\varphi = 3.0$	0.00	0.00	0.07	0.36	0.57
**Scenario 2**	**Marginal recommendation**	$\varphi = 0.1$	0.02	0.22	0.62	0.14	0.00
		$\varphi = 3.0$	0.02	0.25	0.60	0.13	0.00
	**Dose-agnostic recommendation**	$\varphi = 0.1$	0.01	0.21	0.60	0.17	0.00
		$\varphi = 3.0$	0.02	0.20	0.63	0.15	0.00
**Scenario 3**	**Marginal recommendation**	$\varphi = 0.1$	0.15	0.63	0.22	0.00	0.00
		$\varphi = 3.0$	0.16	0.64	0.20	0.00	0.00
	**Dose-agnostic recommendation**	$\varphi = 0.1$	0.14	0.63	0.22	0.01	0.00
		$\varphi = 3.0$	0.15	0.62	0.23	0.00	0.00
**Scenario 4**	**Marginal recommendation**	$\varphi = 0.1$	0.01	0.11	0.40	0.42	0.06
		$\varphi = 3.0$	0.01	0.13	0.44	0.38	0.03
	**Dose-agnostic recommendation**	$\varphi = 0.1$	0.01	0.09	0.34	0.46	0.10
		$\varphi = 3.0$	0.01	0.10	0.42	0.41	0.06
**Scenario 5**	**Marginal recommendation**	$\varphi = 0.1$	0.75	0.24	0.00	0.00	0.00
		$\varphi = 3.0$	0.74	0.26	0.00	0.00	0.00
	**Dose-agnostic recommendation**	$\varphi = 0.1$	0.73	0.26	0.01	0.00	0.00
		$\varphi = 3.0$	0.73	0.26	0.00	0.00	0.00
**Scenario 6**	**Marginal recommendation**	$\varphi = 0.1$	0.04	0.28	0.56	0.12	0.00
		$\varphi = 3.0$	0.04	0.30	0.53	0.12	0.00
	**Dose-agnostic recommendation**	$\varphi = 0.1$	0.03	0.23	0.56	0.17	0.00
		$\varphi = 3.0$	0.04	0.23	0.57	0.15	0.00
**Scenario 7**	**Marginal recommendation**	$\varphi = 0.1$	0.05	0.29	0.55	0.12	0.00
		$\varphi = 3.0$	0.05	0.34	0.52	0.09	0.00
	**Dose-agnostic recommendation**	$\varphi = 0.1$	0.04	0.26	0.53	0.17	0.00
		$\varphi = 3.0$	0.05	0.29	0.56	0.10	0.00
**Scenario 8**	**Marginal recommendation**	$\varphi = 0.1$	0.19	0.61	0.20	0.00	0.00
		$\varphi = 3.0$	0.20	0.66	0.15	0.00	0.00
	**Dose-agnostic recommendation**	$\varphi = 0.1$	0.17	0.61	0.21	0.01	0.00
		$\varphi = 3.0$	0.20	0.63	0.16	0.00	0.00
**Scenario 9**	**Marginal recommendation**	$\varphi = 0.1$	0.22	0.60	0.18	0.00	0.00
		$\varphi = 3.0$	0.23	0.58	0.19	0.00	0.00
	**Dose-agnostic recommendation**	$\varphi = 0.1$	0.19	0.59	0.22	0.00	0.00
		$\varphi = 3.0$	0.19	0.58	0.23	0.00	0.00
**Scenario 10**	**Marginal recommendation**	$\varphi = 0.1$	0.20	0.62	0.17	0.00	0.00
		$\varphi = 3.0$	0.22	0.60	0.18	0.00	0.00
	**Dose-agnostic recommendation**	$\varphi = 0.1$	0.20	0.60	0.20	0.00	0.00
		$\varphi = 3.0$	0.21	0.60	0.18	0.00	0.00
**Scenario 11**	**Marginal recommendation**	$\varphi = 0.1$	0.75	0.24	0.01	0.00	0.00
		$\varphi = 3.0$	0.75	0.25	0.00	0.00	0.00
	**Dose-agnostic recommendation**	$\varphi = 0.1$	0.74	0.25	0.01	0.00	0.00
		$\varphi = 3.0$	0.74	0.26	0.00	0.00	0.00
**Scenario 12**	**Marginal recommendation**	$\varphi = 0.1$	0.74	0.25	0.01	0.00	0.00
		$\varphi = 3.0$	0.77	0.22	0.00	0.00	0.00
	**Dose-agnostic recommendation**	$\varphi = 0.1$	0.75	0.25	0.01	0.00	0.00
		$\varphi = 3.0$	0.77	0.22	0.00	0.00	0.00

Table S3 Proportion of MTD selection recommendations for each dose under 12 random scenarios for PRO-CRM with marginally and jointly calibrated priors with C-DLT and P-DLT correlation parameters $\varphi \in \{0.1, 3.0\}$.

Randomisation Stage

For $\mathcal{A}_n = \{d : \hat{\pi}_{C,d} \leq p_C\}$, at interim n where $\hat{\pi}_{C,d} = u_d^{\hat{\lambda}}$ is estimated similarly to (13), dose recommendations initially follow an adaptive randomisation routine with patients allocated to dose d with probability R_d where,

$$R_d = \frac{\hat{\pi}_{E,d}}{\sum_{d \in \mathcal{A}_n} \hat{\pi}_{E,d}}. \tag{18}$$

Within our simulation study, the first 25% of patients are allocated to doses with probabilities proportional to the estimated efficacy at each dose.

Maximisation Stage

Following this run-in, doses are recommended such that,

$$d^* = \arg\max_{d \in \mathcal{A}} \hat{\pi}_{E,d}. \tag{19}$$

5.2 | Choice of prior for ζ

In the original paper, Wages and Tait use a $\zeta \sim N(0, 1.34)$ prior. In this paper, we use an alternative prior $\zeta \sim HN(0, 0.1)$ where HN indicates a Half-Normal distribution. As $\exp(\zeta) > 0$, no matter the choice of prior, the choice of ζ does not alter the dose-response relationship for each skeleton. At the maximisation stage of the trial, accurate estimation of the probability of response is not required for decision-making as it is only the *choice* of skeleton k^* which informs the next dose decision. Accordingly, the prior for ζ should be chosen to facilitate selection of the dose-response relationship (skeleton) rather than be chosen to best estimate response rate for each dose. The recommended prior on $\zeta \sim N(0, 1.34)$ is broad, often grossly overestimating or underestimating efficacy estimates in comparison to the calibrated efficacy skeletons. This limits the variability among the efficacy skeletons which characterise each dose-response relationship.

Our choice of $\zeta \sim HN(0, 0.1)$ ensures that a priori estimates of ζ remain close to the existing skeleton. Since $\exp(\zeta) > 1$ for $\zeta \sim HN(0, 0.1)$, our elicited prior cannot overestimate efficacy. As we use the original skeletons to calibrate dose-agnostic priors, this ensures that ζ cannot subsequently over-inflate the skeleton when estimating $w(k|\mathcal{D}_n)$. In Figures S1 and S2 we demonstrate the impact of priors $\zeta \sim N(0, 1.34)$ and $\zeta \sim HN(0, 0.1)$ on the prior probability of efficacy for each dose under all considered skeletons and $w(k|\mathcal{D}_n)$. In this scenario, all doses are equally effective and thus the true dose-response skeleton is q_6. Following response observations for the first cohort of patients, the posterior probabilities of efficacy for each skeleton k are presented. When $\zeta \sim HN(0, 0.1)$, the estimated probability of efficacy for dose d ($z_{d,k}^{\exp(\hat{\zeta})}$) is more clearly differentiable for each skeleton compared to when $\zeta \sim N(0, 1.34)$, please see Figures S1a and S2a. This increased variability enables the model to better differentiate between skeletons, supporting the identification of the best efficacious dose and plateau point. This is clearly illustrated in Figures S1b and S2b. When the model is run with prior $\zeta \sim HN(0, 0.1)$, the model has greater success identifying the true skeleton, with that confidence increasing as more patients are enrolled onto the trial. Conversely, with $\zeta \sim N(0, 1.34)$, the model struggles to identify the true skeleton, instead assigning similar posterior probabilities to all skeletons. As a result, dose recommendations when $\zeta \sim N(0, 1.34)$ are primarily driven by the identification of the highest safe dose rather than by accurately identifying the true dose-response relationship.

As a Half-Normal prior cannot estimate the true probability of response larger than that which the skeleton defines, it is crucial to distinguish between priors used for *dose allocation* and those used for *estimating the true efficacy* of a dose. While a Half-Normal prior is well-suited for identifying the true dose-response relationship for a treatment (essential for optimal patient allocation within the Eff+Tox-CRM), it may not be best suited for estimating the true probability of response when the true probability exceeds the pre-defined skeleton estimate. In the use case presented in this paper, where priors are chosen with the aim of optimising the probability of correct selection for the chosen method, the Half-Normal prior is therefore the preferred choice.

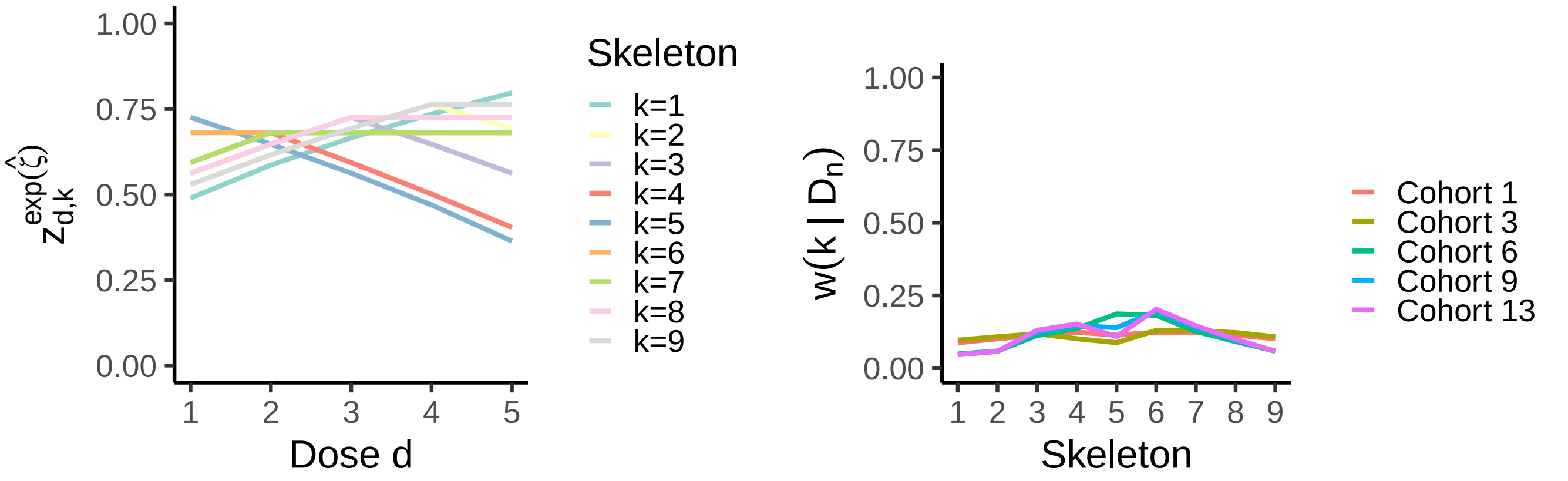

(a) Posterior probability of efficacy for each skeleton k for each dose following the observations of the first cohort.

(b) Posterior probability each skeleton is true for each skeleton k following the observations of cohort 1, 3, 6, 9 and 13 where the true skeleton is q_6.

Figure S1 Hypothetical dose-finding trial using Eff+Tox-CRM with DLT rates (0.05, 0.1, 0.2, 0.28, 0.5) and efficacy rates (0.6, 0.6, 0.6, 0.6, 0.6). A total of 39 patients are treated in cohorts of 3.

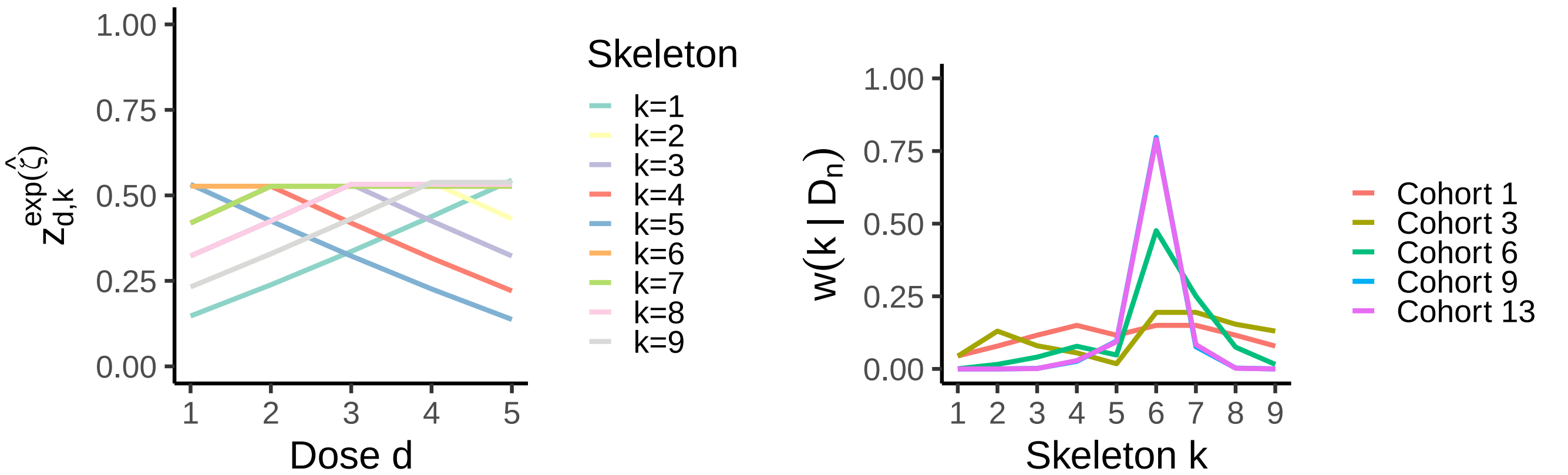

(a) Posterior probability of efficacy for each skeleton k for each dose following the observations of the first cohort.

(b) Posterior probability each skeleton is true for each skeleton k following the observations of cohort 1, 3, 6, 9 and 13 where the true skeleton is q_6.

Figure S2 Hypothetical dose-finding trial using Eff+Tox-CRM with DLT rates (0.05, 0.1, 0.2, 0.28, 0.5) and efficacy rates (0.6, 0.6, 0.6, 0.6, 0.6). A total of 39 patients are treated in cohorts of 3.

6 | EFF+TOX-CRM ENSEMBLE SIMULATION SCENARIOS

In Table S4 we present the ensemble simulation scenarios investigated in the Eff+Tox-CRM simulation study. We investigate 6 C-DLT rate scenarios and 9 response rate scenarios, with 54 (6×9) scenarios investigated in total.

7 | SUPPLEMENTARY EFF+TOX-CRM SIMULATION RESULTS

This section provides further simulation studies beyond those presented in the main manuscript for Eff+Tox-CRM. In Table S5 we present the mean number of DLTs and efficacious responses observed per trial across all 5,000 simulation runs with 12 random simulation scenarios in instances where priors are marginally calibrated or jointly calibrated to be dose-agnostic.

C-DLT rate ensemble scenarios					Response rate ensemble scenarios				
Dose 1	Dose 2	Dose 3	Dose 4	Dose 5	Dose 1	Dose 2	Dose 3	Dose 4	Dose 5
0.05	0.10	0.12	0.15	0.20	0.63	0.63	0.63	0.63	0.63
0.10	0.12	0.15	0.20	0.25	0.63	0.50	0.38	0.25	0.13
0.12	0.15	0.20	0.25	0.40	0.50	0.63	0.63	0.63	0.63
0.15	0.20	0.25	0.40	0.55	0.50	0.63	0.50	0.38	0.25
0.20	0.25	0.40	0.55	0.60	0.38	0.50	0.63	0.63	0.63
0.25	0.40	0.55	0.60	0.70	0.38	0.50	0.63	0.50	0.38
					0.25	0.38	0.50	0.63	0.63
					0.25	0.38	0.50	0.63	0.50
					0.13	0.25	0.38	0.50	0.63

Table S4 Ensemble simulation scenarios for Eff+Tox-CRM.

Whilst the main manuscript evaluates the performance of Eff+Tox-CRM when priors are calibrated with $\alpha_l = 1.17$, in this section we evaluate the operating characteristics for an Eff+Tox-CRM trial design for priors where $\alpha_l \in \{1.03, 1.17, 1.88, 2.51, 3.89\}$. When $\alpha_l = 1.03$, the standard deviation of PCS when one dose is best is 0.06 with a mean PCS of 0.49 across all scenarios. When $\alpha_l = \{1.88, 2.51, 3.89\}$, the performance metrics are similar, with the standard deviation of PCS when one dose is best remaining at 0.08 and the mean PCS across all scenarios at approximately 0.45.

In Figure S3 we demonstrate the proportion of patients dosed correctly, underdosed, and overdosed for each choice of α_l across 12 random scenarios. Whilst standard deviation of PCS and mean PCS are similar for different choices of α_l, there are scenario-specific performance differences for different choices of α_l. For example, in Scenario B utilising priors calibrated with $\alpha_l = 3.89$ greatly increases the proportion of patients overdosed when dose 2 is the true best dose. As such, care must be taken when selecting the value of α_l to ensure that the priors support sufficient exploration of doses whilst maintaining appropriate control of overdosing as determined by the trialists.

	**Mean number of C-DLTs per trial**		**Mean number of efficacy responses per trial**	
	Marginal	Dose-agnostic	Marginal	Dose-agnostic
**Scenario A [Doses 1-5 best dose]**	3.22	4.09	24.48	24.23
**Scenario B [Dose 2 best dose]**	3.51	4.07	21.18	20.18
**Scenario C [Doses 3-5 best dose]**	5.10	5.68	20.25	20.96
**Scenario D [Dose 5 best dose]**	5.78	6.64	13.36	15.98
**Scenario E [Doses 2-4 best dose]**	5.72	6.38	21.94	22.01
**Scenario F [Doses 3-4 best dose]**	6.10	6.78	19.40	19.89
**Scenario G [Dose 3 best dose]**	7.26	7.55	17.62	17.21
**Scenario H [Dose 3 best dose]**	7.59	7.99	14.22	14.37
**Scenario I [Dose 2 best dose]**	7.54	7.30	17.52	16.12
**Scenario J [Dose 2 best dose]**	7.79	7.63	14.44	13.54
**Scenario K [Dose 2 best dose]**	8.05	7.91	10.97	10.66
**Scenario L [Dose 2 best dose]**	8.18	7.91	7.17	7.15

Table S5 Mean number of C-DLTs and efficacy responses per Eff+Tox-CRM trial for 5,000 simulations for marginally calibrated and dose-agnostic priors.

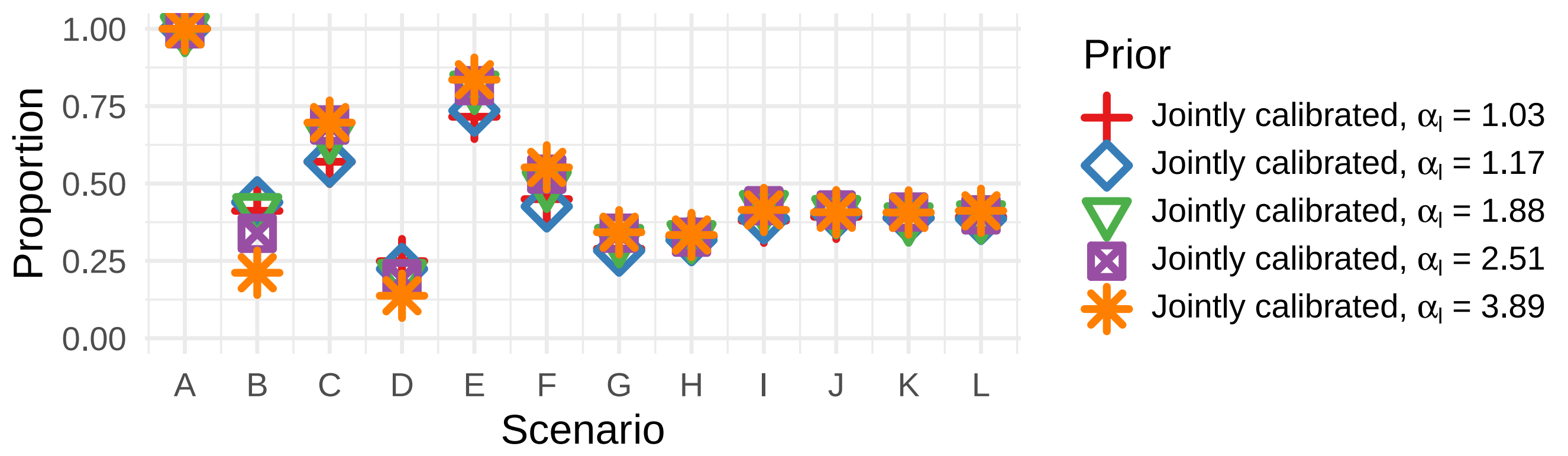

(a) Mean proportion of patients treated at best dose(s).

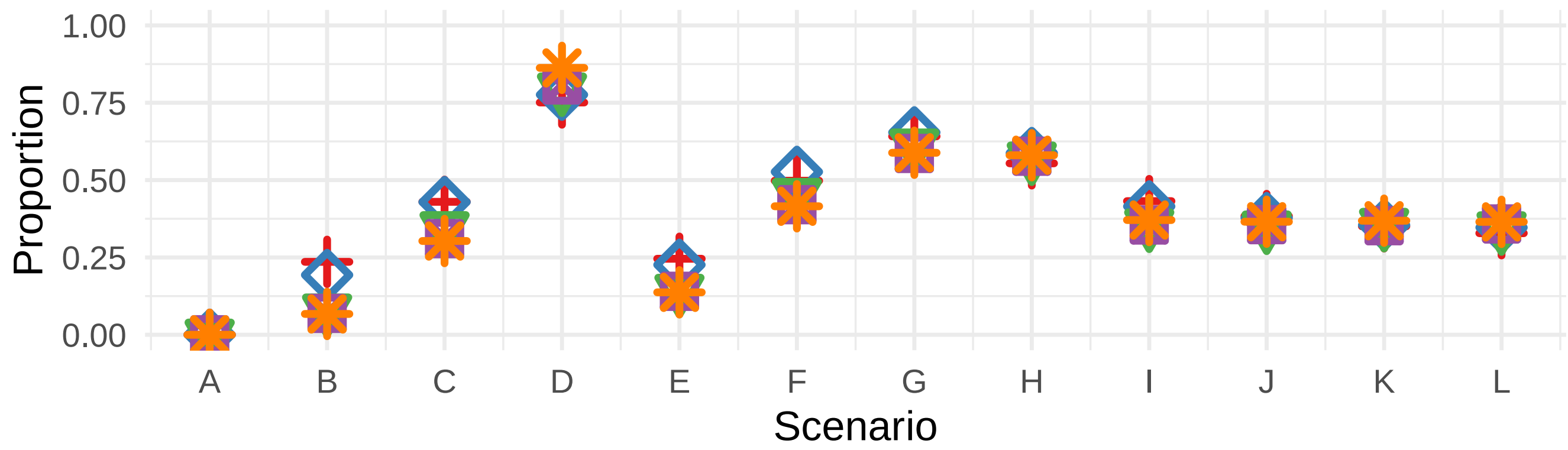

(b) Mean proportion of patients underdosed below best dose(s).

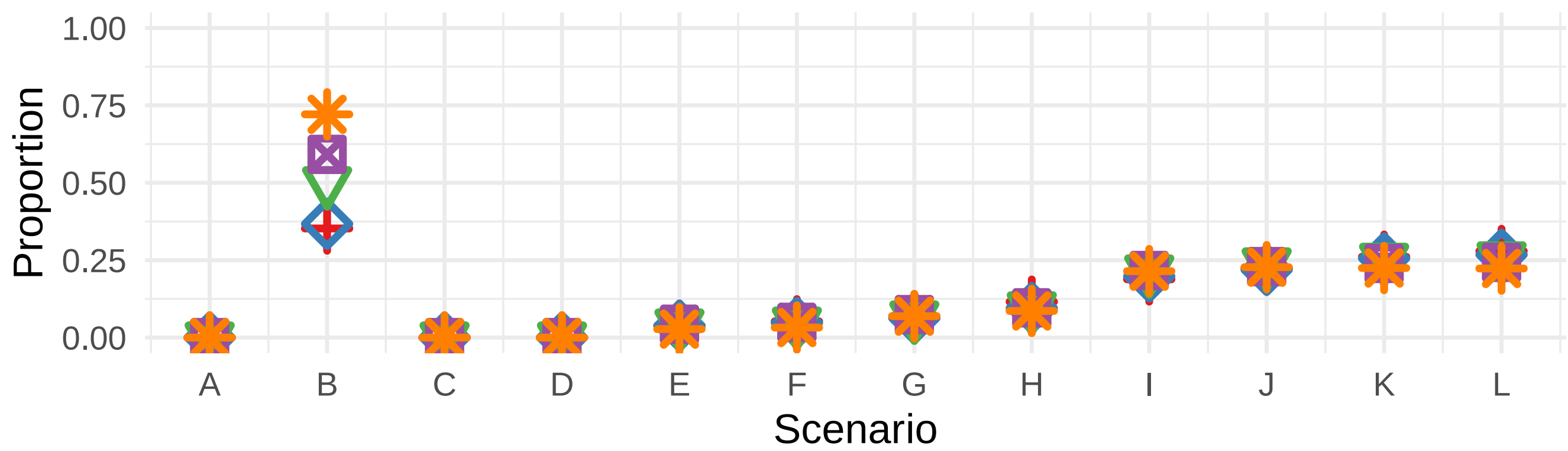

(c) Mean proportion of patients overdosed above best dose(s).

Figure S3 Mean proportion of patients treated at the best dose(s), underdosed, and overdosed across 5,000 simulations for 12 scenarios when priors are jointly calibrated for different choices of α_l.

8 | CALIBRATING SKELETONS FOR EFF+TOX-CRM TRIALS

In this section we provide an exemplar case study to demonstrate how to calibrate C-DLT and efficacy skeletons for the Eff+Tox-CRM trial design. We evaluate the operating characteristics for the design under three skeletons with indifference intervals $\delta = 0.03, 0.05,$ and 0.07 respectively. Once the skeleton has been calibrated accordingly, we then calibrate parameter priors according to any elicited a priori beliefs as per the main manuscript. We then evaluate trial design performance for each skeleton and parameter prior tuning using an ensemble simulation approach.

Consider skeletons calibrated with indifference interval $\delta = 0.05$. Whilst it is very straightforward to calibrate the C-DLT skeleton, calibrating the efficacy skeletons relies on generating specific dose-efficacy patterns. We first determine skeleton q_1 with indifference interval δ, target rate 0.6 and a priori best dose, and dose 5 as the a priori best dose. Note, the target rate is traditionally used to define a target DLT rate, whilst in this case we utilise the target to define the maximum anticipated efficacy rate. Subsequent skeletons q_k are created by modifying q_1 to follow the dose-response patterns to be investigated. In Table S6 we detail the calibrated skeleton using this example.

**d**	**1**	**2**	**3**	**4**	**5**	**d**	**1**	**2**	**3**	**4**	**5**
u_d	0.08	0.16	0.25	0.35	0.46	q_5	0.60	0.49	0.37	0.26	0.15
q_1	0.15	0.26	0.37	0.49	0.60	q_6	0.60	0.60	0.60	0.60	0.60
q_2	0.26	0.37	0.49	0.60	0.49	q_7	0.49	0.60	0.60	0.60	0.60
q_3	0.37	0.49	0.60	0.49	0.37	q_8	0.37	0.49	0.60	0.60	0.60
q_4	0.49	0.60	0.49	0.37	0.26	q_9	0.26	0.37	0.49	0.60	0.60

Table S6 Skeleton for C-DLT and efficacy probabilities obtained using `getprior()` function in `dfcrm` package in R and $\delta_C = 0.05$.

The efficacy and clinician-DLT skeletons serve distinct roles within the trial design. The clinician-DLT skeleton is primarily involved in estimating the C-DLT rate, while the efficacy skeleton is mainly used to differentiate among the various dose-response relationships. In Figure S4 we present PCS performance for the marginal and dose-agnostic priors for 12 random scenarios when clinician-DLT and response skeletons are calibrated using varying half-widths δ.

For indifference intervals $\delta = 0.05$ and 0.07, dose-agnostic priors maintain their superiority in scenarios where larger doses are best, yielding less variability in PCS across all simulations in line with results of the main manuscript. In line with the recommendations of Lee and Cheung, similarly extensive evaluations should be conducted in practice to ensure that optimal PCS may be achieved for any joint-outcome dose-finding trial design.

9 | PRIOR CALIBRATION WHEN μ_ϕ IS NOT AVAILABLE IN CLOSED FORM

When μ_ϕ is not available in closed form, we estimate it empirically via Monte Carlo simulation. Specifically, for a given choice of $\boldsymbol{\phi}$, we generate N independent samples $\boldsymbol{\theta}_1, \dots, \boldsymbol{\theta}_N \sim f(\boldsymbol{\theta}; \boldsymbol{\phi})$ and compute the corresponding dose recommendations $\nu(\boldsymbol{\theta}_1), \dots, \nu(\boldsymbol{\theta}_N)$. The dose-space distribution is then estimated by

$$\tilde{\mu}_\phi(d) = \frac{1}{N} \sum_{i=1}^{N} \mathbb{1}\{\nu(\boldsymbol{\theta}_i) = d\}, \tag{20}$$

where $\mathbf{1}\{A\}$ denotes the indicator function. As in the main manuscript, we use the Kullback-Leibler divergence as a calibration criterion to identify suitable hyperparameters $\boldsymbol{\phi}^* \in \Phi$ whose induced dose-space distribution is close to target prior distribution P as per,

$$\sum_{d \in D} \tilde{\mu}_{\boldsymbol{\phi}}(d) \log \frac{\tilde{\mu}_{\boldsymbol{\phi}}(d)}{P(d)}. \tag{21}$$

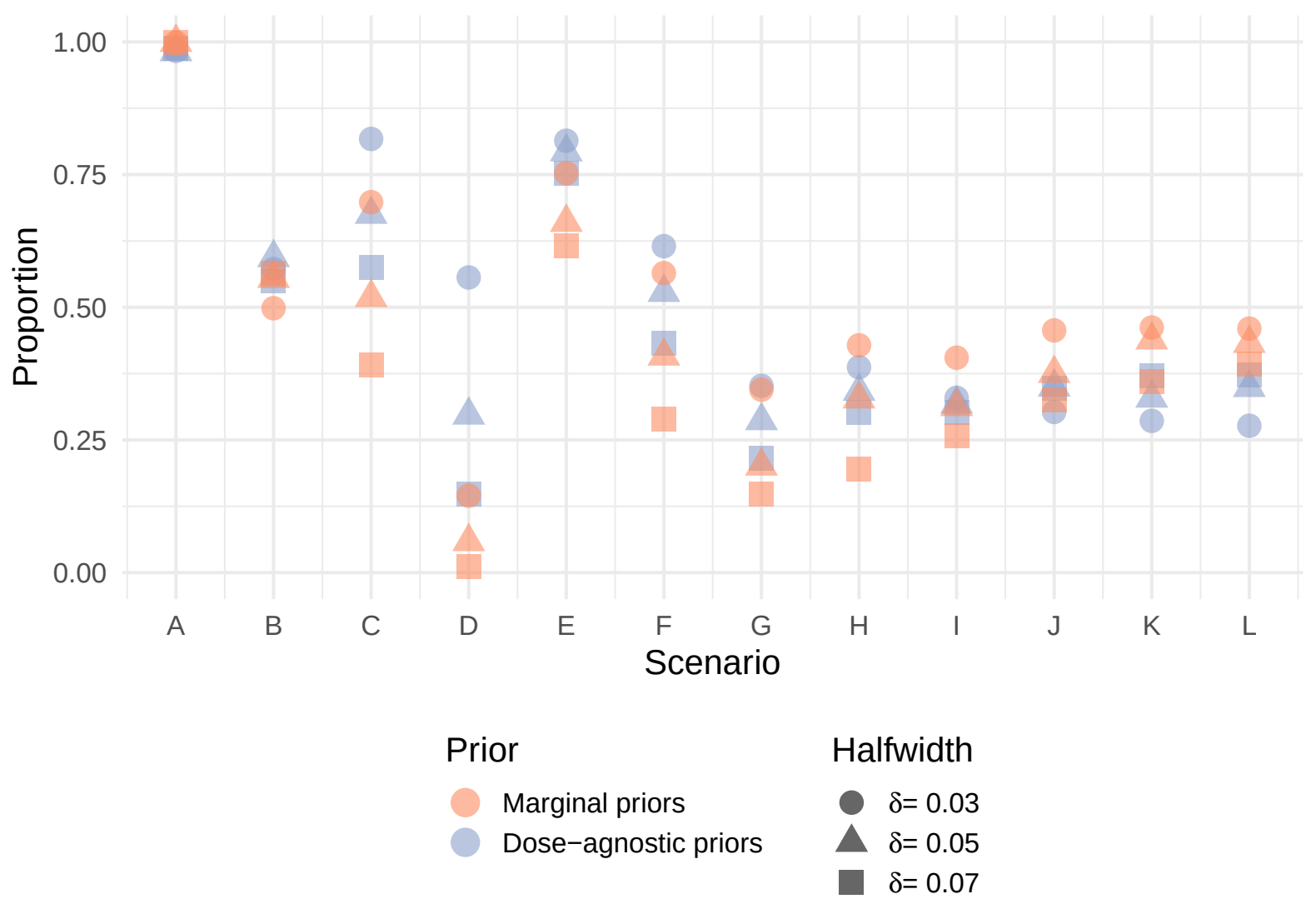

Figure S4 Proportion of best dose(s) selection recommendations for each dose under 12 scenarios using `getprior()` function in `dfcrm` with three different half-widths with priors which are calibrated as per the modified marginal and jointly calibrated approach.

We illustrate this approach using the EffTox trial design.[1] The EffTox design is implemented following Brock et al,[2] so that the decision criterion can be written as,

$$\nu(\boldsymbol{\theta}) = \min \arg \max_{d \in \mathcal{D}} U_d(\boldsymbol{\theta}), \tag{22}$$

where,

$$U_d(\boldsymbol{\theta}) = 1 - \left[\left(\frac{1 - \pi_{E,d}(\boldsymbol{\theta})}{0.6} \right)^{2.07} + \left(\frac{\pi_{T,d}(\boldsymbol{\theta})}{0.7} \right)^{2.07} \right]^{0.48}, \tag{23}$$

where $\pi_{T,d}(\boldsymbol{\theta})$ and $\pi_{E,d}(\boldsymbol{\theta})$ denote the model-based probabilities of toxicity and response for dose $d \in \mathcal{D}$ respectively. Figure S5 shows the performance of the calibration criterion in four prior scenarios for an EffTox trial with four doses based on 5,000 Monte Carlo samples. Across all scenarios, the KL divergence between the calibrated empirical prior distribution $\tilde{\mu}_{\phi}$ and the target prior distribution is at most 0.008.

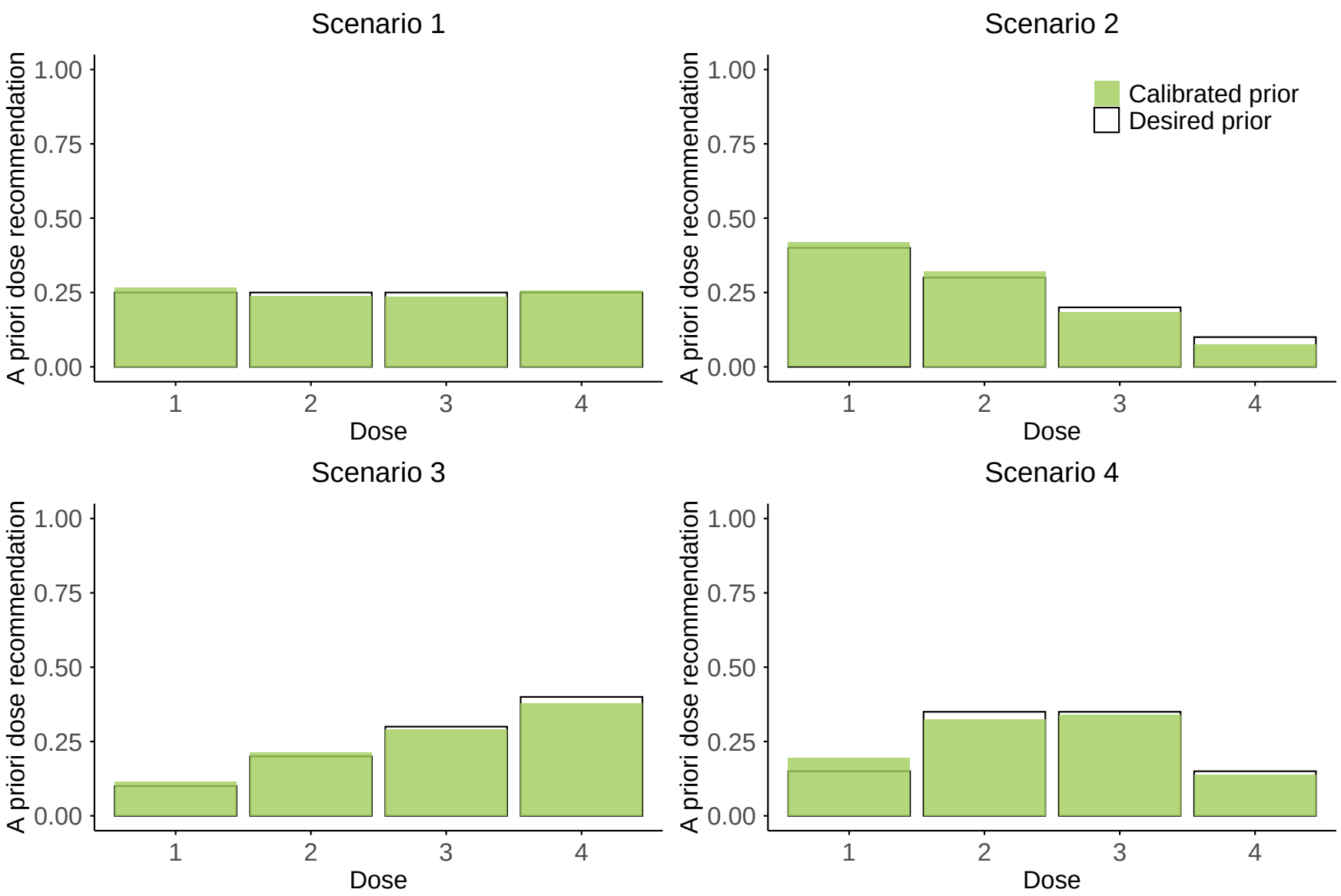

Figure S5 *A priori* dose recommendations under four potential prior-calibration scenarios for the EffTox[1] trial design, with the true scenario shown in black and the calibrated empirical prior recommendation shown in green.

References

[1] Peter F Thall and John D Cook. "Dose-finding based on efficacy–toxicity trade-offs". In: Biometrics 60.3 (2004), pp. 684–693.

[2] Kristian Brock et al. "Implementing the EffTox dose-finding design in the Matchpoint trial". In: BMC medical research methodology 17.1 (2017), p. 112.
